# Ligand induced switching of the band alignment in aqueous synthesized CdTe/CdS core/shell nanocrystals

**DOI:** 10.1038/s41598-019-44787-y

**Published:** 2019-06-06

**Authors:** Brener R. C. Vale, Rafael S. Mourão, Jefferson Bettini, José C. L. Sousa, Jefferson L. Ferrari, Peter Reiss, Dmitry Aldakov, Marco A. Schiavon

**Affiliations:** 1 Grupo de Pesquisa em Química de Materiais - GPQM, Departamento de Ciências Naturais, Universidade Federal de São João del-Rei, Campus Dom Bosco, Praça Dom Helvécio, 74, CEP, 36301-160 São João del-Rei, Minas Gerais Brazil; 2 Univ. Grenoble Alpes, CNRS, CEA, INAC-SyMMES-STEP, 38000 Grenoble, France; 3 Laboratório Nacional de Nanotecnologia, Centro Nacional de Pesquisa em Energia e Materiais, Campinas, São Paulo Brazil

**Keywords:** Fluorescence spectroscopy, Scanning probe microscopy, Nanoparticles, Quantum dots

## Abstract

CdTe/CdS core/shell quantum dots (QDs) are formed in aqueous synthesis via the partial decomposition of hydrophilic thiols, used as surface ligands. In this work, we investigate the influence of the chemical nature (functional group and chain length) of the used surface ligands on the shell formation. Four different surface ligands are compared: 3-mercaptopropionic acid, MPA, thioglycolic acid, TGA, sodium 3-mercaptopropanesulfonate, MPS, and sodium 2-mercaptoethanesulfonate, MES. The QD growth rate increases when the ligand aliphatic chain length decreases due to steric reasons. At the same time, the QDs stabilized with carboxylate ligands grow faster and achieve higher photoluminescence quantum yields compared to those containing sulfonate ligands. The average PL lifetime of TGA and MPA capped QDs is similar (≈20 ns) while in the case of MPS shorter (≈15 ns) and for MES significantly longer (≈30 ns) values are measured. A detailed structural analysis combining powder X-ray diffraction, and X-ray photoelectron spectroscopy (XPS) indicates the existence of two novel regimes of band alignment: in the case of the mercaptocarboxylate ligands the classic type I band alignment between the core and shell materials is predominant, while the mercaptosulfonate ligands induce a quasi-type II alignment (MES) or an inverted type I alignment (MPS). Finally, the effect of the pH value on the optical properties was evaluated: using a ligand excess in solution allows achieving better stability of the QDs while maintaining high photoluminescence intensity at low pH.

## Introduction

Quantum dots (QDs) are colloidal semiconductor particles on the nanoscale that have attracted great attention in the last three decades. Their main feature is the strong dependence of the band gap on the particle size due to the quantum confinement effect^1–4^. QDs have unique optical properties such as symmetric and sharp emission spectra with high photoluminescence quantum yields (up to virtually 100% depending on size, composition and synthetic route), broad absorption spectra with high extinction coefficients (10^4^–10^5^ M^−1^ cm^−1^), and valence and conduction band levels (VB and CB, respectively) compatible with the CB level of TiO_2_ and ZnO, which are the most common metal oxide semiconductors used in photovoltaic devices^5,6^. For these reasons, QDs have been studied in many applications such as LEDs^7^, photovoltaic devices^5,8–11^, biomarkers^12^, and fluorescence sensors^13–15^.

In colloidal QD synthesis, surface ligands (SL) serving to avoid aggregation, give stability and enable surface functionalization^16^. Our group has previously investigated different SL in the synthesis of CdTe QDs, such as 3-mercaptopropionic acid (MPA), L-glutathione, thioglycolic acid (TGA), and 1-thioglycerol^17^. In another study we have established that short molecules bearing just one functional group (e.g. propionic acid, propanethiol, propylamine, and mercaptoethylamine) are not suitable to stabilize the QDs even though the QDs capped with the latter have some colloidal stability just above a pH value of 6^18^. By FT-IR spectroscopy it was inferred that for the CdTe QDs capped with the above mentioned SL the functional group responsible for metal coordination is the thiol (S-H)^17,18^ according to the Pearson’s principle^18,19^. Therefore, for the synthesis of QDs in aqueous media, there is a requirement of at least two functional groups, one to coordinate to the metal surface sites of the semiconductor and the other to interact with the solvent. Moreover, this latter group should be charged to maintain the electrostatic repulsion between QDs to keep the colloidal stability^18^.

The synthesis of QDs is governed by two major processes - nucleation and growth. Nucleation is driven by the difference in the free energy between the crystalline phase and the solution, and therefore by the gain in chemical potential (free energy to bonding formation) and increase in the total surface energy (correction to incomplete saturation of surface bonding). Growth, in turn, can be divided into two steps: i) the diffusion of monomers to the nanocrystals surface, and ii) the reaction between free monomers and the crystal surface^1^. SL bind to the QD surface and create a dynamically bound stabilizing layer that allows the addition of monomers. The rate of monomer delivery is affected by the nature and binding of the SL, which therefore affect the growth rate of QDs, their size, and shape^20,21^. For example, the interaction between the ligand’s functional group with different crystalline facets can guide nanocrystals’ growth in one crystallographic direction^20^. The structure of the ligand (chain length, branching and functional groups) also determines its steric hindrance, which is related to the rate of monomer diffusion through the ligand layer^20^. While the influence of SL on the physicochemical properties of QDs is extensively studied for organic synthesis, much fewer works are available for the aqueous medium. Thus, the influence of the SLs on the electronic levels and the trap states are not discussed.

The aim of this work is to study the effect of the functional group and the chain length of SL on the structure and optical properties of colloidal water-soluble CdTe QDs. QDs stabilized with four different SL were synthesized: two bearing –COOH groups (MPA and TGA), and two bearing –SO_3_ groups (sodium 3-mercaptopropanesulfonate (MPS) and sodium 2-mercaptoethanesulfonate (MES). SL with carboxylic groups are the most studied in water-soluble QDs synthesis, including their behavior in aqueous media and well-established functionalization reactions^17,18,22,23^. However, to our best knowledge there are no studies of MPS for CdTe synthesis even though this group has low pKa values, which opens a potential of their use in a larger pH range. We have studied the photoluminescence properties of the obtained QDs as a function of the chemical structure of the SL, as well as their capacity of forming CdS layers on the CdTe core. Finally, the stability of the QDs was evaluated as a function of the pH, and the behavior of the sulfonate-bearing SL was studied and compared to the carboxylate SL.

## Experimental Section

### Chemicals

The following chemicals were used in the synthesis of CdTe QDs: Cadmium chloride monohydrate (CdCl_2_ H_2_O 99%), hydrochloric acid (HCl, 37% v/v) and thioglycolic acid (TGA, 98%) were acquired from Vetec. Tellurium powder (200 mesh, 99.8%), 3-mercaptopropionic acid (MPA, 99%), sodium 3-mercaptopropanesulfonate (MPS, 90%), sodium 2-mercaptoethanesulfonate (MES, 98%), sodium borohydride (NaBH_4_, 98%) were obtained from Sigma-Aldrich. Sodium hydroxide (NaOH, 98%) was acquired from Neon. Ultrapure water (electric conductivity <1 μS/cm) was used in all syntheses of QDs and all chemicals were used without previous purification.

### Synthesis of CdTe quantum dots

The synthesis of CdTe QDs was realized in two steps. The first step comprises the preparation of a tellurium precursor solution (NaHTe) while the second one consists of the injection of this solution into the cadmium-SL solution (SL: MPA, TGA, MPS, or MES)^17^. NaHTe solution was prepared using 0.4 mmol of tellurium (Te) powder and 0.8 mmol of sodium borohydride (NaBH_4_), which were dissolved in 10 mL of water under inert atmosphere. After that, this solution was heated to 80 °C with constant stirring for 30 minutes. The reaction of the tellurium precursor can be written according to Eq. 1 ^24^.

1 $${\rm{4}}\,{{\rm{NaBH}}}_{{\rm{4}}({\rm{s}})}+{\rm{2}}\,{{\rm{Te}}}_{({\rm{s}})}+{\rm{7}}\,{{\rm{H}}}_{{\rm{2}}}{{\rm{O}}}_{({\rm{l}})}\to {\rm{2}}\,{{\rm{NaHTe}}}_{({\rm{aq}})}+{{\rm{Na}}}_{{\rm{2}}}{{\rm{B}}}_{{\rm{4}}}{{\rm{O}}}_{{\rm{7}}({\rm{aq}})}+{\rm{14}}\,{{\rm{H}}}_{{\rm{2}}({\rm{g}})}$$

**Figure 1:**
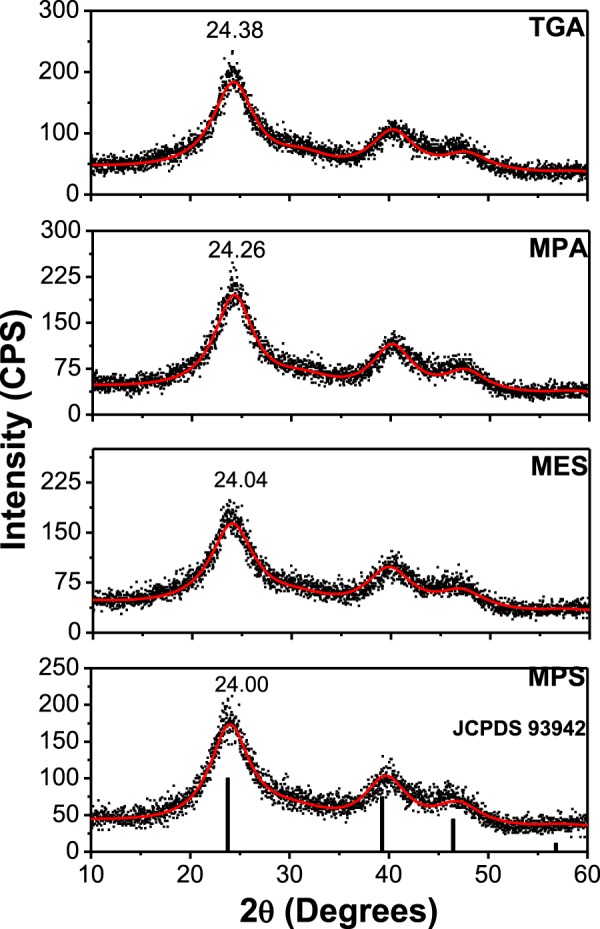
Powder X-ray diffractograms of CdTe QDs capped with different surface ligands. The red line represents the fitting of the data. The vertical lines identify the diffraction pattern of bulk CdTe (23.77°; 39.36°, and 46.42°) (JCPDS #93942).

The second step of the synthesis was carried out employing 0.8 mmol of CdCl_2_.H_2_O, 2.8 mmol of SL (ratio SL:Cd^2+^ was 3.5) both dissolved in 160 mL of water under constant stirring. The pH of this solution was adjusted to 10.0 by the addition of 1.0 M NaOH, and subsequently, it was refluxed under inert atmosphere. Finally, 8.0 mL of the NaHTe solution prepared in the first step was injected into the solution containing Cd^2+^/SL. This moment was taken as the onset of synthesis of CdTe QDs. In Fig. 1S (see supporting information) a presentation of the synthetic process is shown. Aliquots were taken from the reaction medium in regular time intervals, and their absorption and emission spectra were registered at room temperature. For XPS and XRD measurements a post-preparative precipitation method was applied for the preparation of QD powders, using acetone as a nonsolvent in order to destabilize the colloidal solution. Then, the precipitate was collected by centrifugation at 5000 rpm for 5 minutes. Finally, the powder was dried at 40 °C^24^.

### Sample preparation for pH test

The preparation of Britton and Robinson’s buffer solution consists of mixing acetic, boric and phosphoric acids at the same concentrations of 0.2 M. The choice of this buffer was motivated by the broad range of its applicability to cover a full pH range in water^25^. To adjust the pH 2.0 M NaOH was added. To keep the ionic strength constant, 1.0 M KCl was also added to all solutions with the adjusted pH. After 240 minutes of synthesis, the dispersion of CdTe/SL QDs was diluted directly. The optical density of the colloidal solutions at the excitation wavelength (355 nm) was kept below 0.1, with the objective of avoiding reabsorption, inner filter effects, and coagulation of QDs at low pH. Two approaches were applied, with and without SL excess. For the experiment set with SL in excess their concentration was 0.0175 M.

### Characterization

Absorption spectra were acquired employing a spectrophotometer (Shimadzu, UV-2450/2550), with a spectral resolution of 1.0 nm, and a slit width of 1.0 nm, in the 200–700 nm region. Emission spectra in the steady state were registered employing a spectrofluorometer (Shimadzu, RF-5301 PC) equipped with a 150 W Xenon lamp. The spectral resolution utilized was 1.0 nm, the excitation and emission slits were both set to 3.0 nm. The excitation wavelength was at 355 nm. Measurements of time-resolved photoluminescence spectra to obtain PL decay curves were registered using a spectrofluorometer (Horiba Jobin Yvon, Fluorolog-3) equipped with a nano-LED as pulsed excitation source, with a peak at 340 nm, and peak lifetime less than 0.8 ns. The method utilized was Time-correlated Single Photon Counting (TCSPC). The repetition rate was 1.00 MHz and a range of measurements was of 200 ns in 3650 channels with a resolution of 5.49 × 10^−2^ ns channel^−1^. All measurements of absorption and emission were performed in quartz cuvettes (Hellma) of 10.00 mm optical path length. Powder X-ray diffraction (XRD) was performed on a diffractometer (XRD-6000, Shimadzu) using CuKα radiation. The diffraction angle was varied from 10 to 80 degrees (2 theta) with a rate of 1° min^−1^. Energy dispersive X-ray spectroscopy (EDX) spectra were recorded on a ZEISS Ultra 55+ scanning electron microscope equipped with an EDX probe (acceleration tension: 20 kV; working distance: 7 mm). For sample preparation, a concentrated colloidal solution of QDs in water was drop-cast on the surface of a cleaned silicon substrate. Measurements of X-ray photoelectron spectroscopy (XPS) were conducted using a Versa Probe spectrometer (ULVAC-Phi) with a Al Kα monochromatic source (1486.6 eV). The core level peaks were recorded with constant pass energy of 23.3 eV. The XPS spectra were fitted with CasaXPS software using Shirley background and a combination of Gaussian (70%) and Lorentzian (30%) distributions. Binding energies are referenced with respect to the adventitious carbon (C 1s BE = 284.6 eV). The TEM and STEM images were acquired using a microscope JEOL JEM 2100 equipped with a camera TVIPS F416. The samples analyzed by XRD, XPS, EDX, TEM, and STEM were QDs obtained with a synthesis time of 240 minutes.

## Results and Discussion

The synthesis of CdTe QDs could be successfully realized with all proposed ligands. Powder XRD analyses of the final products confirm that the crystal structure of the obtained QDs can be assigned to the cubic zinc-blende phase (JCPDS 93942)^26,27^. Figure 1 shows the diffractograms of CdTe QDs capped with different SL.

The diffraction peaks for all QDs studied are shifted to higher angles with respect to bulk CdTe, indicating a reduced lattice parameter. Liu^28^ and Rogach^26^ observed similar behavior for CdTe QDs capped with thiol-based SL. They assigned this behavior to a partial hydrolysis of thiol molecules at prolonged synthesis times leading to sulfur incorporation on the surface of the QDs, forming a core/shell structure of CdTe/CdS. Since all samples analyzed by XRD in this study were obtained after 240 minutes of synthesis, this argument is in accordance with our experimental data. The diffractograms were fitted by Rietveld refinement employing the software MAUD^29^ to obtain the main diffraction peaks position with precision (Fig. 1). It can be seen that CdTe/TGA and CdTe/MPA QDs are more displaced to higher angles than the samples prepared with MPS and MES. This result indicates that the former samples present a more pronounced formation of CdS resulting in a thicker shell in the CdTe/CdS core/shell structure. This effect will be confirmed by the XPS results presented below.

**Figure 2:**
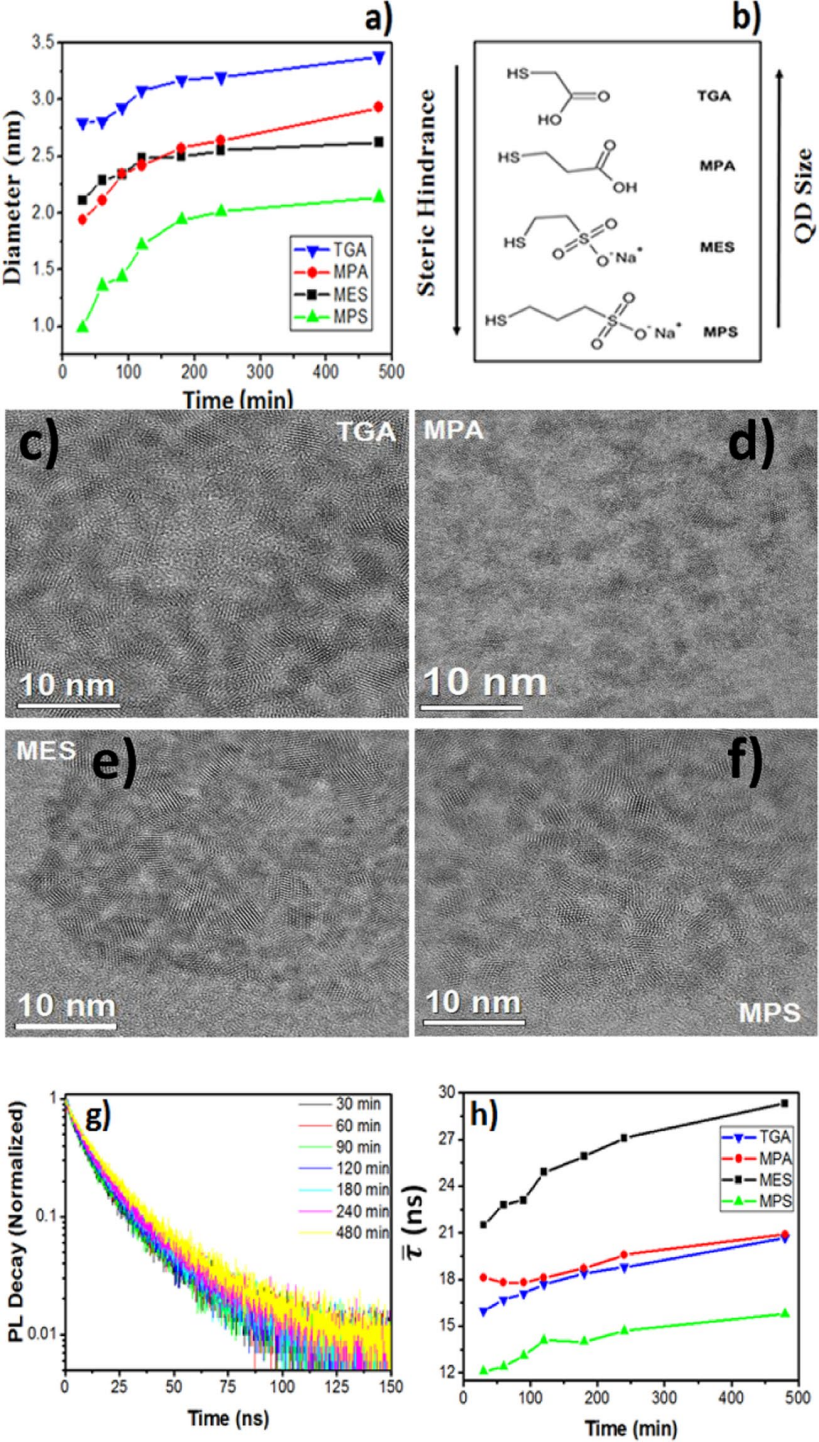
(**a**) Average diameter of the QDs *versus* synthesis time. (**b**) Relationship between QDs size and steric hindrance of SL used in the CdTe synthesis. TEM images of CdTe QDs of (**c**) TGA, (**d**) MPA, (**e**) MES, (**f**) MPS ligands. (**g**) PL decay curves of CdTe/MPA QDs. (**h**) PL average lifetime of CdTe QDs as a function of synthesis time.

**Figure 3:**
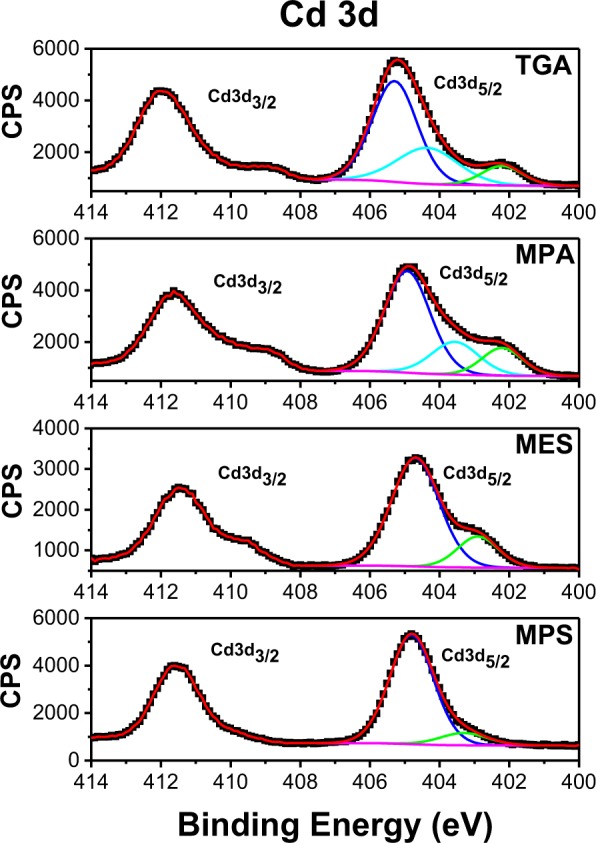
High-resolution XPS spectra of Cd 3d for CdTe QDs capped with different ligands.

Figure 2S (see supporting information) shows photoluminescence (PL) emission spectra of CdTe QDs capped with different SL, and the absorption spectra are presented in Fig. 3S. A red shift with increasing synthesis time can be observed in all cases due to the weakening of the quantum confinement effect with the increase of particle size^4^.

**Figure 4:**
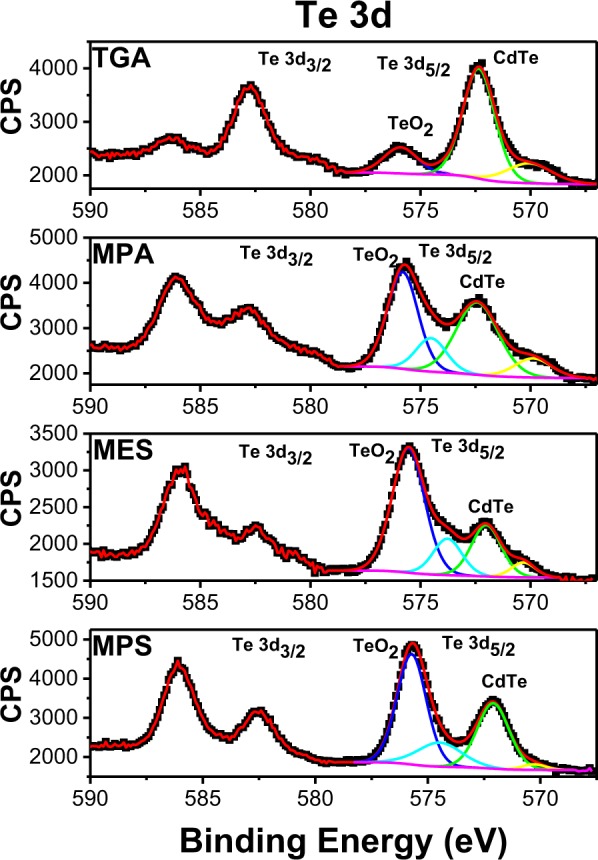
High-resolution XPS spectra of Te 3d for CdTe QDs capped with different ligands.

The analysis of the maximum wavelength, for both emission and absorption as a function of synthesis time (see Fig. 4S) allowed following the growth rate of the QDs. The average diameter obtained was found through Equation 2 ^30^:

2 $$D=(9.8127\times {10}^{-7}){\lambda }^{3}-(1.7147\times {10}^{-3}){\lambda }^{2}+(1.0064)\lambda -(194.84)$$

where λ is the maximum absorption wavelength at the first excitonic band of the QDs, which was achieved from an adjusted Gaussian function on the experimental data. The average diameter of CdTe QDs calculated as a function of synthesis time was compared for the four systems (Fig. 2a).

Comparing the growth rate of QDs capped with different SL, it can be observed that QDs containing sulfonate groups (MPS, MES) grow slower compared to those with the carboxylate group (MPA, TGA). As the sulfonate group is more bulky than the carboxylate one, the diffusion of the monomers to the surface of the QDs in the former case is more difficult. It was also observed that CdTe QDs capped with SL containing three carbon atoms (MPA and MPS) in the chain remain at smaller sizes than those with just two carbons (TGA and MES), likely also due to the steric hindrance reasons (Fig. 2a). Yo *et al*. have previously found similar results for MPA- and TGA-capped PbS QDs^31^, while Morris *et al*. observed that increasing the SL chain length slows down the growth process in CdSe QDs synthesized in organic medium^20^.

**Figure 5:**
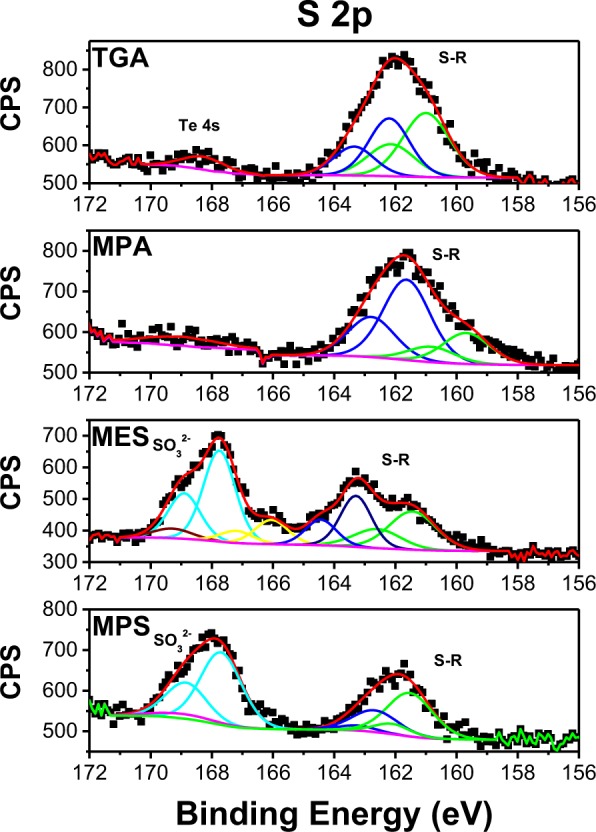
High-resolution XPS spectra of S 2p of CdTe QDs capped with different ligands.

**Figure 6:**
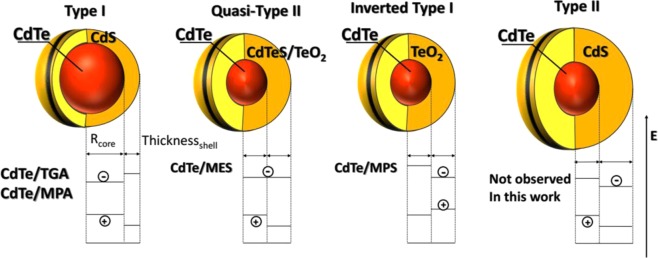
Structure of different types of core-shell: Type I, quasi-Type II and Type II.

**Figure 7:**
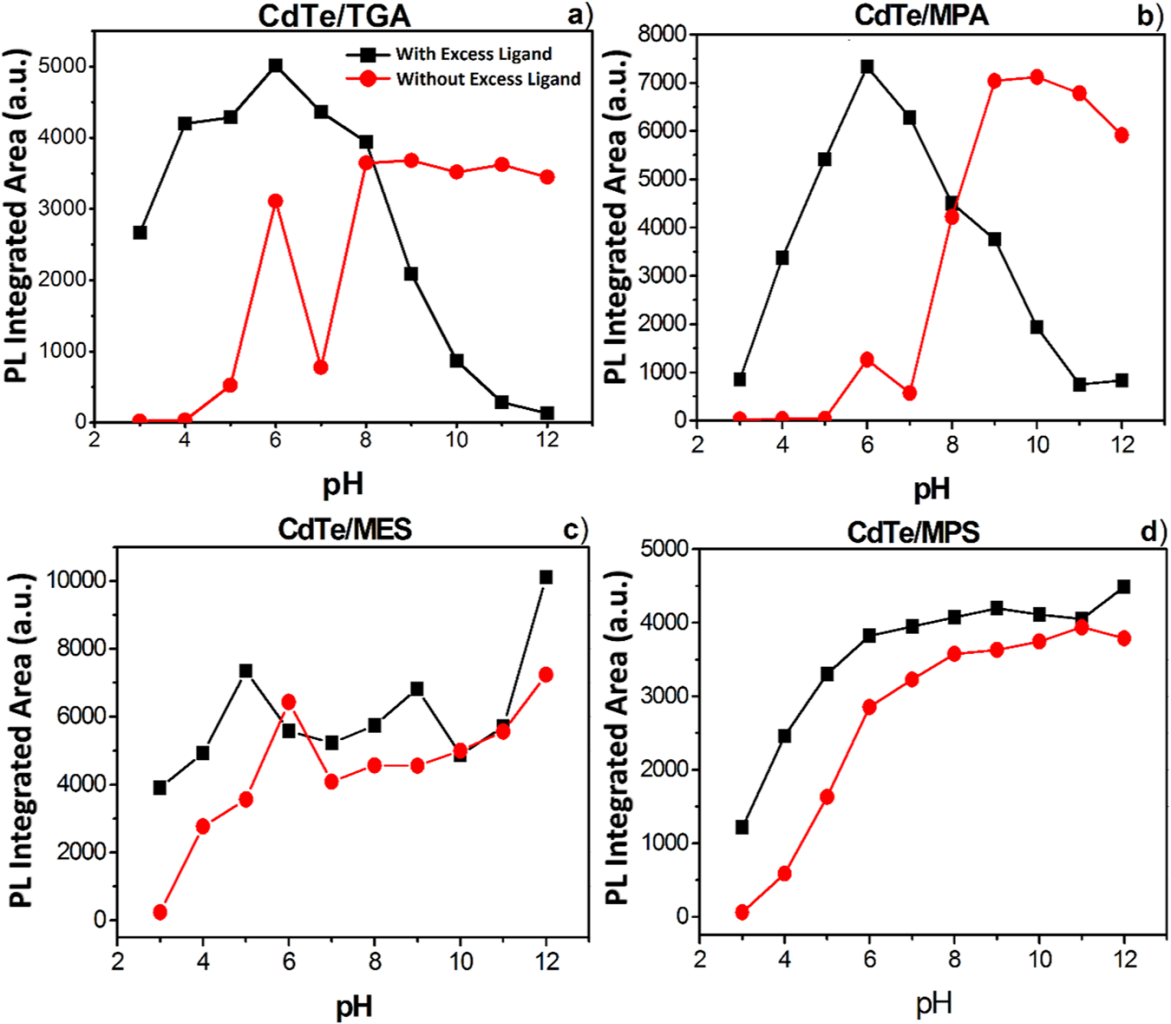
Integrated PL of (**a**) CdTe/TGA, (**b**) CdTe/MPA, (**c**) CdTe/MES and (**d**) CdTe/MPS QDs as a function of pH.

Figure 2c–f (and Figs 5S and 6S) show the TEM images of CdTe QDs after 240 minutes of synthesis capped with different SL. The size distribution was calculated for each sample (Fig. 7S). The mean size of QDs obtained by TEM measurements is in good agreement with that calculated by the Equation 2 (Fig. 2a) with systematically slightly larger diameters obtained by the latter. This difference comes from the broad absorption spectra (Fig. 3S), which is related to the broad size distribution and due to shell formation, as it will be discussed in the text.

Figure 2g shows the PL decay curves of CdTe/MPA QDs (the data for the other systems can be found on Fig. 8S). The curves can be fitted perfectly with a biexponential function using Equation 3:

3 $${\rm{I}}({\rm{t}})={{\rm{\alpha }}}_{1}\,\exp \,(\,-\,{\rm{t}}/{{\rm{\tau }}}_{1})+{{\rm{\alpha }}}_{2}\,\exp (\,-\,{\rm{t}}/{{\rm{\tau }}}_{2})$$

where I(t) is the PL intensity at time t, α is the amplitude of the component and τ is the PL lifetime. The values of the fitting of all samples are in the Tables 1S–4S. The average lifetime ($\bar{{\boldsymbol{\tau }}}$) was calculated using Equation 4:

4 $$\bar{\tau }=({{\rm{\alpha }}}_{1}\,{{\rm{\tau }}}_{1}^{2}+{{\rm{\alpha }}}_{2}\,{{\rm{\tau }}}_{2}^{2})/({{\rm{\alpha }}}_{1}\,{{\rm{\tau }}}_{1}+{{\rm{\alpha }}}_{2}\,{{\rm{\tau }}}_{2})$$

The slower decay component (>20 ns) is usually attributed to the recombination of the exciton on the surface of QDs, while the faster decay component is related to intrinsic recombination of states initially populated in the core^32^. Figure 2h shows the average lifetime of QDs capped with different SL. It can be observed that the QDs capped with MPA and TGA have comparable behavior due to the similar structure of the ligands. On the contrary, MES and MPS demonstrate completely different lifetime behavior, which cannot be explained by the chemical structure of the SL, which differ only by one methylene group in the alkyl chain. Moreover, the lifetimes of CdTe/MES QDs were systematically higher than for all other systems.

PL quantum yield measurements throughout the full time of synthesis were carried out for all samples, employing the method proposed by Williams (Fig. 9S)^33–35^. The quantum yields of CdTe QDs capped with the different SL studied at 240 min of synthesis are presented in Table 1.

**Table 1:** Relative concentration calculated from integrated peak intensity ratios extracted from XPS data.

Sample	Te_3d 5/2_ TeO_2_/CdTe^1^	Te_3d 5/2_/Cd_3d 5/2_	Cd_3d 5/2_/S_2p_^2^	Te_3d 5/2_/S_2p_	QY (%)
CdTe/TGA	0.21	0.34	1.58	0.55	23.4 ± 0.8
CdTe/MPA	0.84	0.61	1.69	1.03	18.0 ± 0.4
CdTe/MES	1.46	0.60	0.53	0.32	12.0 ± 0.4
CdTe/MPS	1.57	0.72	1.28	0.92	1.6 ± 0.1

^1^TeO_2_/CdTe refers to ratio of bands related to TeO_2_ (Te^4+^ binding to O) and CdTe (Te^2−^ binding to Cd^2+^); ^2^SO_3_^2−^ peaks were not taken into account.

As can be seen, the QDs capped with carboxylate-based SL and MES reached higher quantum yield values compared to the MPS capped ones. The lifetime and quantum yield results can be understood as the superposition of two effects: increase in the QDs size and coating of the core with a CdS shell. Both effects likely contribute to the decrease of the density of traps on the surface of the QDs, increasing the PL quantum yield. As the smaller QDs have higher surface-to-volume ratio, they are more susceptible to surface defects creating trap states for charge carriers. With the increase of QD size this ratio decreases and so does the density of defects. The second possible reason for the increase of the PL quantum yield is related to ligand degradation. As mentioned above, the thiol group can release sulfide ions in the reaction medium upon prolonged synthesis times. These ions can react with Cd^2+^ ions in solution forming a thin shell of CdS passivating the core of CdTe QDs and improving the quantum yield.

The chemical composition of QDs was assessed using X-ray photoelectron spectroscopy (XPS). Figure 11S shows a survey XPS spectrum of all systems studies in this work. The presented survey spectrum is similar to other studies reported in the literature for CdTe QDs^36,37^. Besides cadmium and tellurium from the core, carbon, sulfur, and oxygen present in the SL were detected. For this study, we focused on Cd 3d, Te 3d, and S 2p. Figure 3 shows high-resolution XPS spectra of Cd 3d for CdTe QDs capped with the different SL.

In the Cd 3d high-resolution XPS spectra of CdTe QDs several peaks were observed, at around 405, 404, and 402 eV. Two first peaks can be due to CdTe according to previous studies^36–38^. Borchert *et al*. used XPS with tunable synchrotron radiation excitation and discriminated the peaks of surface- (at higher energies) and core-localized (at lower energies) cadmium telluride^36^. In our case the peaks at 404 and 405 eV could indeed correspond to the core- and surface-localized Cd because for smaller sizes the ratio of surface atoms becomes considerable (e.g. for MPS-capped CdTe QDs surface atoms represent 63% of all QD atoms, see supporting information for more details). However, the exact interpretation of Cd 3d_5/2_ peaks in similar materials is complicated because the signals of CdTe, CdS, CdO and CdTeO_3_ are generally poorly resolved. The shoulder at low energies observed only for TGA and MPA-capped QDs corresponds likely to the beam damage of the QD surface indicating their lower stability under such X-ray irradiation.

On the Te 3d XPS spectra of CdTe QDs four peaks are generally observed (Fig. 4). The peak with the highest energy at around 576 eV is attributed to Te^4+^ from TeO_2_ or CdTeO_3_ occurring on the surface of QDs as a result of partial oxidation, while the peak at 572 eV is ascribed to Te^2−^ from CdTe^37^. The smaller peak at 574 eV likely comes from the surface-localized under-passivated Te^2−^ as suggested by Borchert *et al*.^36^, while the remaining minor peak at *ca.* 570 eV is possibly a result of beam damage of the sample similar to the one observed for Cd 3d. The relative intensities of these peaks differ depending on the ligand employed, indicating a variation of the concentration of these species and thus of the relative oxidation degree of the QDs. The corresponding areas of these bands were integrated and compared (Table 1).

As can be seen from Table 1, the smallest ratio TeO_2_/CdTe was obtained for CdTe/TGA followed by CdTe/MPA. This result shows that TGA and MPA SL are more efficient to prevent the QDs from surface oxidation compared to sulfonate ligands. Moreover, this result goes in line with the previous observation of the higher PL quantum yield found for these ligands (Table 1).

In Fig. 5 we present the S 2p XPS spectra of CdTe QDs capped with the different SL. For sulfonate-containing QDs (MPS and MES) three or four main peaks are present, each of them occurring as a doublet corresponding to S 2p_3/2_ and S 2p_1/2_, while for CdTe/TGA and CdTe/MPA only two peaks are observed systematically. The band situated at around 168 eV in the case of MES and MPS ligands is attributed to the oxidized S^6+^ of the sulfonate group, while two doublets present for all the samples at around 161 and 162 eV are due to sulfide S^2**−**^ ^39,40^ and thiolate Cd-S-R ions^41,42^. In order to distinguish between them, an additional experiment has been performed: the samples have been subjected to prolonged X-ray beam exposure and the S 2p regions before and after exposure were compared (Fig. 12S). The shape of the signal has changed with the intensity of 162 eV peak decreased after the exposure. Previously, this has been explained by the presence of the thiolates partially degrading with time under the beam^43^. This allowed to ascribe the sulfur peak at 162 eV to the thiolates and the one at 161 eV to the S^2**−**^.

Sulfur is an element of much lower atomic number compared to cadmium and tellurium resulting in a significant shift of binding energy according to its chemical environment. Thus, sulfur bound to oxygen appears clearly at greater energy than when it is bound to another element. In the case of the tellurium, it is possible to differentiate between the TeO_2_ and CdTe primarily because of the significant change in the Te oxidation state (+4 vs −2). This behavior is not observed for cadmium in CdTe and CdO due to its higher atomic weight compared to sulfur and the same oxidation state in the two compounds, therefore the effect of chemical environment is less pronounced and it is more challenging to differentiate between CdTe and CdS.

The relative concentration of Cd, Te and S have been calculated and compared for the CdTe QDs with different SL studied (cf. Table 1). Thus, for CdTe/TGA the ratio between Te and Cd is the lowest, while the size and PL quantum yield are the largest. For CdTe/MPS QDs having the highest Te/Cd ratio the trend is opposite. For MPA and MES capped QDs having similar particle size, PL quantum yield and Te/Cd ratio are also close. Therefore, these results show that there is a clear correlation between the stoichiometry of aqueous CdTe QDs and their PL quantum yield and size.

Another parameter evaluated from XPS data was the contents of sulfur vs. cadmium for the different studied systems. As can be seen from the Table 1, for QDs with TGA and MPA Cd/S values are very close, likely indicating that the hydrolysis of the thiol group forming sulfides takes place to the same extent for both SL. As the difference of the optical and structural properties between these two QDs is not large, it can be inferred that the shell thickness of CdS in the two systems is similar. This conclusion is corroborated by the PL lifetimes, which are very close for the two types of QDs (cf. Fig. 2h).

The highest Cd/S ratio was observed for CdTe/MPS QDs. Together with the previously discussed highest Te/Cd ratio, this indicates that the surface of CdTe/MPS QDs is sulfur poor and tellurium rich, namely in form of tellurium oxide, resulting in deep trap states on the surface formed by TeO_2_. This hypothesis explains the lowest PL quantum yield (QY) and the shortest PL lifetime presented by these QDs, and also why the absorption spectra shift with the refluxing time and the emission spectra are not (see Fig. 4Sa,b). CdTe/MES QDs, in turn, present the lowest Cd/S ratio, suggesting a thicker CdTeO_2_S shell compared to other QDs studied and accompanied by the longest PL lifetime (see Fig. 2d). Additionally, we have carried out an EDX study, which probes a depth at least 1 µm in the sample. The results are shown in Table S6. In order to be comparable with the EDX results the ratios involving sulfur (Cd/S and Te/S) in XPS results are calculated for the total amount of sulfur in contrast to the Table 1. As can be seen from the table, the ratios obtained by two different methods are mainly comparable and follow the same trends confirming the applicability of the XPS method for the analysis of differently sized QDs in our study.

To better understand the relationship between the CdS shell thickness and the PL lifetime data, we point out that in the case of a thin shell a type I band alignment is expected, keeping the electron-hole pair confined within the CdTe core, which leads to a direct transition^27,40,44^. With increasing CdS shell thickness, a transition to a type II heterostructure occurs, with the CB and VB of the core located above those of the shell, respectively, confining the electron within the shell and the hole within the core, and leading to the a change of the transition to indirect^39,40,44^. For intermediate shell thicknesses, a quasi-type II heterojunction is obtained, characterized by quasi aligned CB energy levels of the core and the shell, while the hole is kept in the core. For this system, the transition feature is between direct and indirect transition. The last configuration is called Inverted type I, when the CB and VB of the shell are located between the CB and VB of the core. In this configuration both electron and hole are located in the shell, obviously yielding a direct transition, similar to the type I configuration. However, the emission in this system is governed by the shell. Once the exciton is on the surface, the PLQY is expected to be low.

The transition from type I to type II behavior, and also that of type I to quasi-type II is influenced by the degree of compression that the core experiences due to the shell, having a smaller lattice parameter. The larger the thickness of the shell is, the higher is the compression and the more the CB of the core is shifted to higher energies. If the core is extremely small and with high density of defects, an inverted type I configuration can occur. The different situations mentioned above are illustrated in Fig. 6.

Different energy levels alignment implies that the structures with the type I and II heterojunctions have completely different properties due to different electronic transitions. These characteristics result in longer PL lifetimes and smaller oscillator strength for the transitions within the type II and quasi-type II structures when compared to the type I and inverted type I. Several studies indicate that core-shell systems with smaller cores are more sensitive to the compression effects compared to the larger ones^27,45^. Thus, because of the short lifetime (~20 ns), large QDs size and intermediate Cd/S ratio of CdTe/MPA and CdTe/TGA QDs, we conclude that the CdTe/CdS core-shell structures formed are of type I. On the other hand, the core-shell structure of CdTe/MES is expected to be a CdTe/Cd(TeO_2_)S quasi-type II due to its smaller size, smaller Cd/S ratio and longer PL lifetime (~30 ns) compared to those for CdTe/MPA and CdTe/TGA QDs. Furthermore, this hypothesis can be corroborated by the XRD results revealing the compression effect: as mentioned above, the (111) diffraction peak of CdTe/MES QDs is less shifted to higher angles despite low Cd/S ratio from the XPS results (Fig. 1). In order to differentiate between type II and quasi-type II system, we have compared their properties. The core-shell type II has longer lifetime (100 *vs* 29 ns) and emission wavelength (650 *vs* 560 nm) then quasi-type II, because the CB of the shell in the former is lower, decreasing the energy of the transition and increases the indirect transition feature. Finally, the core-shell structure of CdTe/MPS is expected to be a CdTe/Cd(TeO_2_) inverted type I due to high degree of oxidation revealed by the XPS results, by the shortest PL lifetime founded (~12 ns), and finally and critical, the behavior of the emission and absorption spectra (see more discussion in the Supporting information, Figs 13S–17S).

In addition, we have observed an asymmetry and broadening in all emission spectra of the samples. Then, we performed a deconvolution process with two or three Gaussian functions in these spectra in order to get more information about the system. The deconvolution results for CdTe/TGA and CdTe/MPA are quite similar with two bands (Figs 13S and 14S), one due to exciton recombination and another one due to shallow trap states, which possibly originate from TeO_2_, as suggested by XPS results. The low energy bands of CdTe/TGA are much less intense and are located at lower energies than those of CdTe/MPA, and both diminish with synthesis time in agreement with the literature data and all results presented here. As a consequence, we hypothesize that higher concentration of the traps increases the energy level of trap states. CdTe/MES PL spectrum also shows two bands (Fig. 15S), however the band located at lower energy is shifted compared to the first one only by 80 meV, while for TGA and MPA ligands the observed shift is of 270 and 200 meV, respectively. On the other hand, the PL signal of CdTe/MPS QDs shows three bands shifted by 75 and 250 meV (Fig. 16S). This energy difference suggests that the type of the traps in TGA and MPA-capped QDs is different from MES ones, while in the case of MPS there are likely two types of trap states. As mentioned before, we can conclude that the CdTe/MPA and CdTe/TGA QDs formed a CdTe/CdS core/shell alignment of type I. In this situation, the energy level of the shell does not contribute to the emission spectra, once it is higher than the bottom of the CB of the core. Because of that, the band located ~200 meV in this configuration can be attributed to emissive trap states. CdTe/MES QDs formed a CdTe/Cd(TeO_2_)S core/shell quasi-type II alignment. Here the CB of the core and shell is quite close, because of that the two bands in the emission spectra can be attributed to the recombination in the core and in the shell. The increase of the amplitude of the longer component in the PL lifetime for CdTe/MES QDs is in accord to our deconvoluted emission spectra, see Fig. 17S. Finally, the three bands observed in CdTe/MPS can be attributed to the recombination in the core, shell, and trap states. It is possible to see that the trap band (~600 nm) practically does not change with increasing synthesis time, and this is coherent with the PLQY behavior. The shell band (~550 nm), increases with the synthesis time, due to the better passivation of the surface. Moreover, we observed that the maximum of the absorption band and emission band of the CdTe/MPS QDs is completely different from the other systems studied here (Fig. 4S). CdTe/TGA QD showed redshift about 40 nm in both absorption and emission spectra, CdTe/MPA ~30 nm, and CdTe/MES ~ 10 nm. However, CdTe/MPS showed ~30 nm in the absorption, and just ~5 nm in the emission spectra. This result is odd, it indicates that the core grown up during the synthesis(absorption shift), however, the emission is not governed only by the core size, but mainly by the shell, which confirms our previous conclusion about the inverted-type I alignment.

As mentioned above, in addition to the CdS shell formation, the XPS results reveal partial oxidation of the QDs, confirmed by the presence of CdO and TeO_2_. The oxide formation can be influenced by two factors: i) the method of tellurium reduction, which can lead to undesired TeO_2_ subproducts, and ii) the pH used during the synthesis. To evaluate the stability of QDs as a function of the pH, a systematic study of integrated PL was performed by varying the pH in the range of 2 to 12 of a buffer solution containing the QDs either simply purified or in excess of SL (Fig. 7). According to Peng *et al*.^46,^ this strategy allows to study the ligands adsorption on the QD surface: under diluted conditions the SL tend to desorb from the surface due to the equilibrium displacement causing the PL quenching. On the contrary, by adding ligand excess the desorption is suppressed^46^.

As can be observed, the results with and without ligand excess differ significantly between the employed ligands. The QDs with ligand excess generally presented higher PL, which persisted even at low pH compared to those without excess ligand. It can indicate a better passivation of the surface due to the high concentration of free ligands in solution. Such high PL intensity of QDs observed at low pH can be important for their application as sensitizers in solar cells: good PL often indicates good QD surface passivation related to fewer surface defects and consequently less charge losses as well as better adsorption of CdTe QDs on typical semiconductors (TiO_2_, ZnO…) used in these cells. In addition, better stability of the QDs at low pH is a considerable advantage for their use for photocatalytic water splitting typically requiring acidic conditions.

The PL behavior of QDs without excess of SL remains stable in the pH range of 8–12. At pH 7 occurs an abrupt decrease for CdTe/MPA and CdTe/TGA QDs, while in the case of CdTe/MES and CdTe/MPS the PL intensity just diminished slightly. At pH 6, for all SL there was a considerable increase in the PL intensity, which can be due to additional sulfurisation reaction on the QDs surface^47^. In addition, in our synthesis conditions, the molar ratio of Cd^2+^: Te^2−^: SL was set to 1:0.5:3.5, which allows the formation of cadmium-ligand complex in solution. Previous optical spectroscopy studies of cadmium-thiol complex demonstrated that its concentration strongly decreases with the decrease of pH and that below pH 4 the complexation does not occur^41^. On the other hand, at low pH thiols are more strongly bound to CdS nanoparticles than to free Cd^2+^ ions^42^. Gao *et al*. conclude that at low pH in solution there are more free ligands than cadmium-ligand complexes, and thus the ligands can be bound to QDs surface more efficiently^16^. Zhang *et al*. evaluated the pH effect on CdTe QDs, analyzed the PL and the ratios of TeO_2_ and sulfur bands by XPS^37^. They have found that the QDs exhibited higher PL and larger S/Te ratio at pH 6 compared to higher pH (1.43 at pH 6.0 vs 0.77 at pH 9) confirming the influence of the pH on the interaction between QDs and SL and its ability to prevent the oxides formation (CdO and TeO_2_).

Under ligand excess conditions, the PL of CdTe/TGA and CdTe/MPA QDs strongly decreases at the pH above 8 (Fig. 7a,b). To explain this behavior, UV-vis absorption spectroscopy of the ligands alone in solution as a function of pH was performed (Fig. 18S). The absorption spectra of TGA and MPA ligands were more sensitive to the increasing pH values indicating that pH strongly influences the electronic environment of SL. The spectral changes are especially pronounced at pH > 8, which coincides with the pKa of the thiol group in organic molecules (about 8.0), suggesting that such behavior can originate from the deprotonation of the thiol group. Moreover, it is interesting to note that the major changes in SL absorption spectra are concomitant with the QD PL decrease indicating that under the ligand excess the PL quenching of CdTe/MPA and CdTe/TGA QDs is closely related to the chemical transformation of the SL.

## Conclusion

In this work we synthesized CdTe QDs capped with four different surface ligands, including previously never studied MPS. The QDs capped with ligands containing the sulfonate group have slower growth rate compared to those containing carboxylate group, likely due to steric hindrance induced by the sulfonate group. Using XPS it was possible to confirm the formation of a thin shell of CdS on CdTe and the partial oxidation of QDs as judged by the peaks of CdO and TeO_2_. The origin of the oxides formation was explained by two related factors in the synthesis method: the pH used for the reduction of tellurium and the employed SL. The observed PL quantum yield and lifetime behavior and additional XRD and XPS measurements provide strong evidence that CdTe/TGA and CdTe/MPA form a type I core-shell heterostructure, CdTe/MES QDs a quasi-type II and CdTe/MPS an inverted type I core-shell structure. Our study further shows that the use of excess ligands in solution yields higher PL even at low pH making this approach appealing for the sensitization of metal oxide substrates with QDs for use in solar energy conversion and in particular in photocatalysis.

## Supplementary information

Supplementary Infomation
