# Supplementary material for: Ligand induced switching of the band alignment in aqueous synthesized CdTe/CdS core/shell nanocrystals
Source: Sci Rep. 2019 Jun 6;9:8332. doi: 10.1038/s41598-019-44787-y (PMC6554334; doi:10.1038/s41598-019-44787-y)
Supplement: Supplementary file 1 — Supplementary Infomation [file 41598_2019_44787_MOESM1_ESM.pdf]

Supplementary information

Ligand induced switching of the band alignment in aqueous synthesized CdTe/CdS core/shell nanocrystals

Brener R. C. Vale[a], Rafael S. Mourão[a], Jefferson Bettini[c], José C. L. Sousa[a], Jefferson L. Ferrari[a], Peter Reiss[b], Dmitry Aldakov[b*], Marco A. Schiavon[a]*

[1]*Grupo de Pesquisa em Química de Materiais - GPQM, Departamento de Ciências Naturais, Universidade Federal de São João del-Rei, Campus Dom Bosco, Praça Dom Helvécio, 74, CEP, 36301-160 - São João del-Rei, Minas Gerais, Brazil.*

.[2]*Univ. Grenoble Alpes, CNRS, CEA, INAC-SyMMES-STEP, 38000 Grenoble, France*

[c] *Laboratório Nacional de Nanotecnologia, Centro Nacional de Pesquisa em Energia e Materiais, Campinas, São Paulo, Brazil.*

*Corresponding Authors email address: dmitry.aldakov@cea.fr; schiavon@ufsj.edu.br

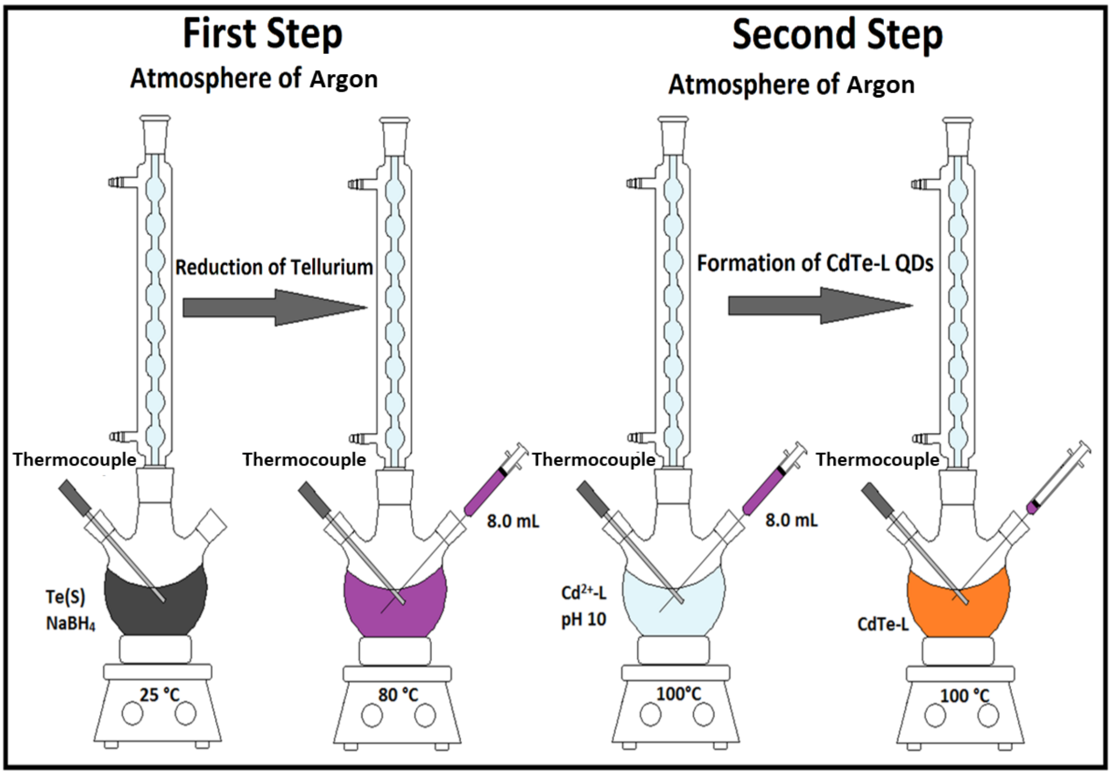

Figure 1S: Illustration of synthetics process of QDs

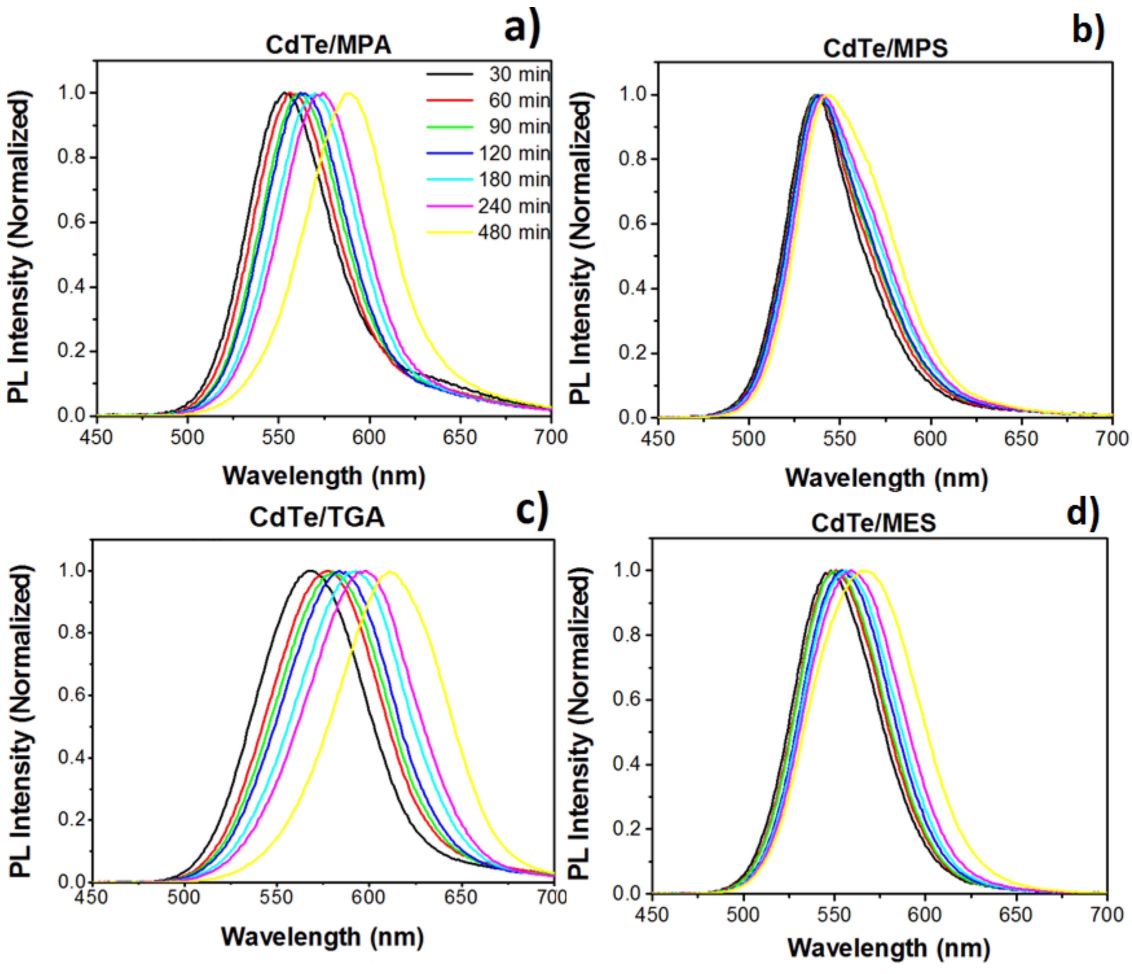

Fig. 2S. Fluorescence spectra of CdTe QDs capped with different ligands (a) MPA, (b) MPS, (c) TGA, and (d) MES.

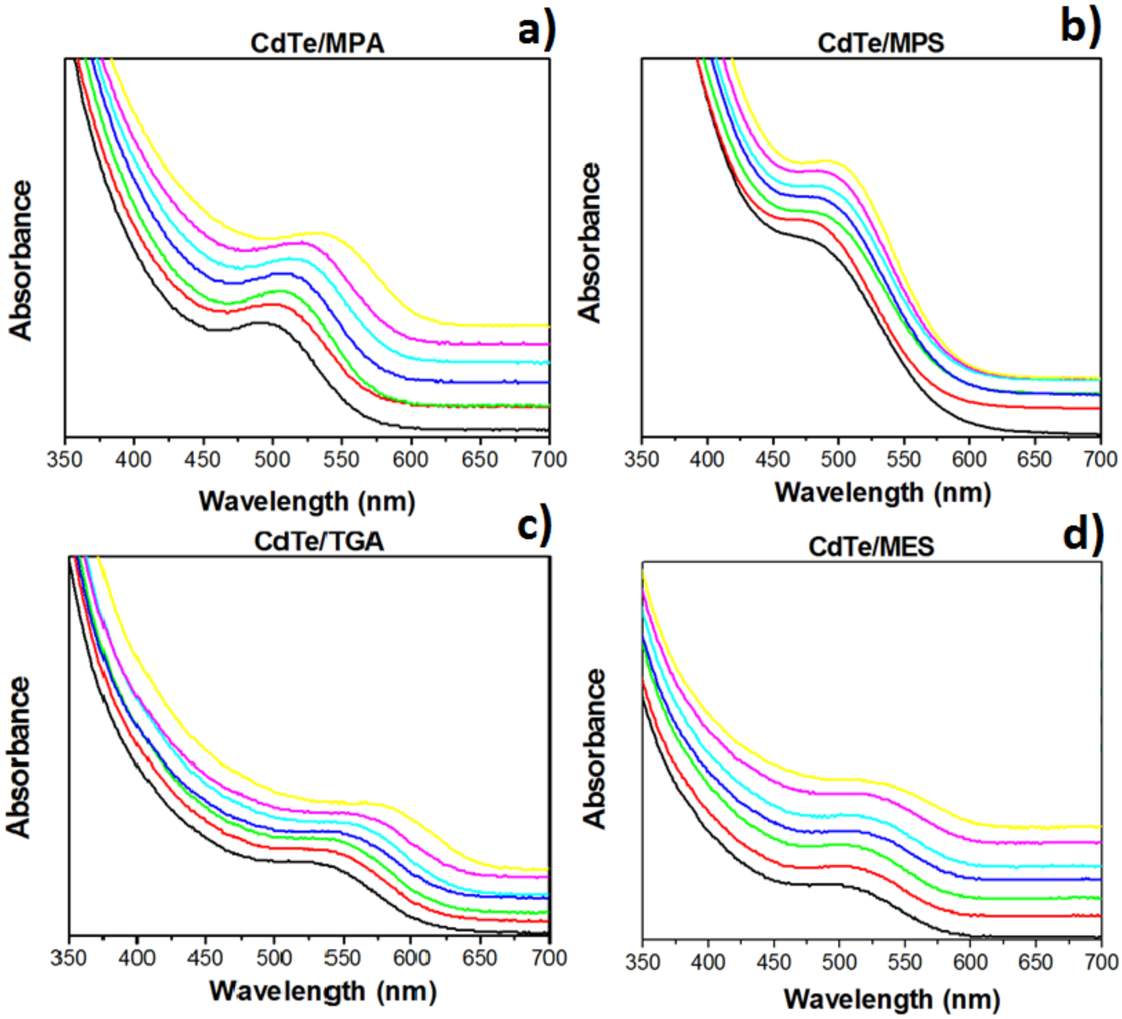

Fig. 3S: absorption spectra of CdTe QDs capped with ligands (a) MPA, (b) MPS, (c) TGA, and (d) MES

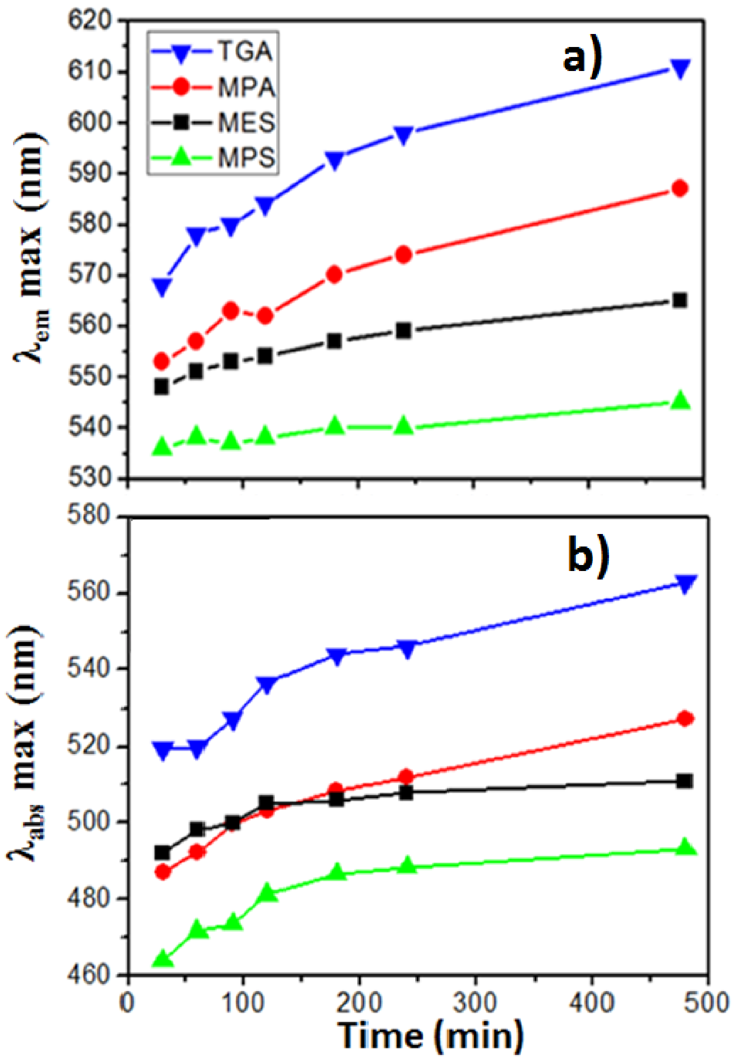

Fig. 4S: a) Emission and b) Absorption wavelength maximum *versus* synthesis time.

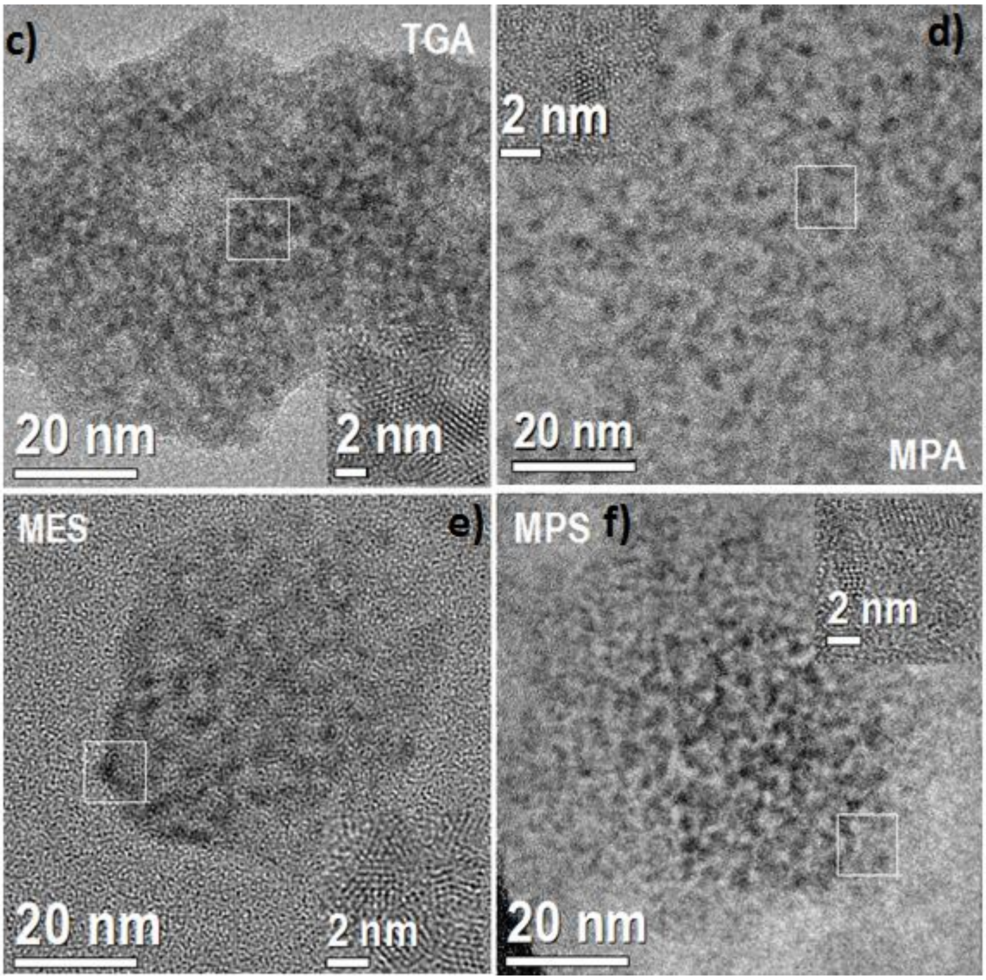

Fig. 5S: TEM images of CdTe QDs capped with different SL (inset HRTEM).

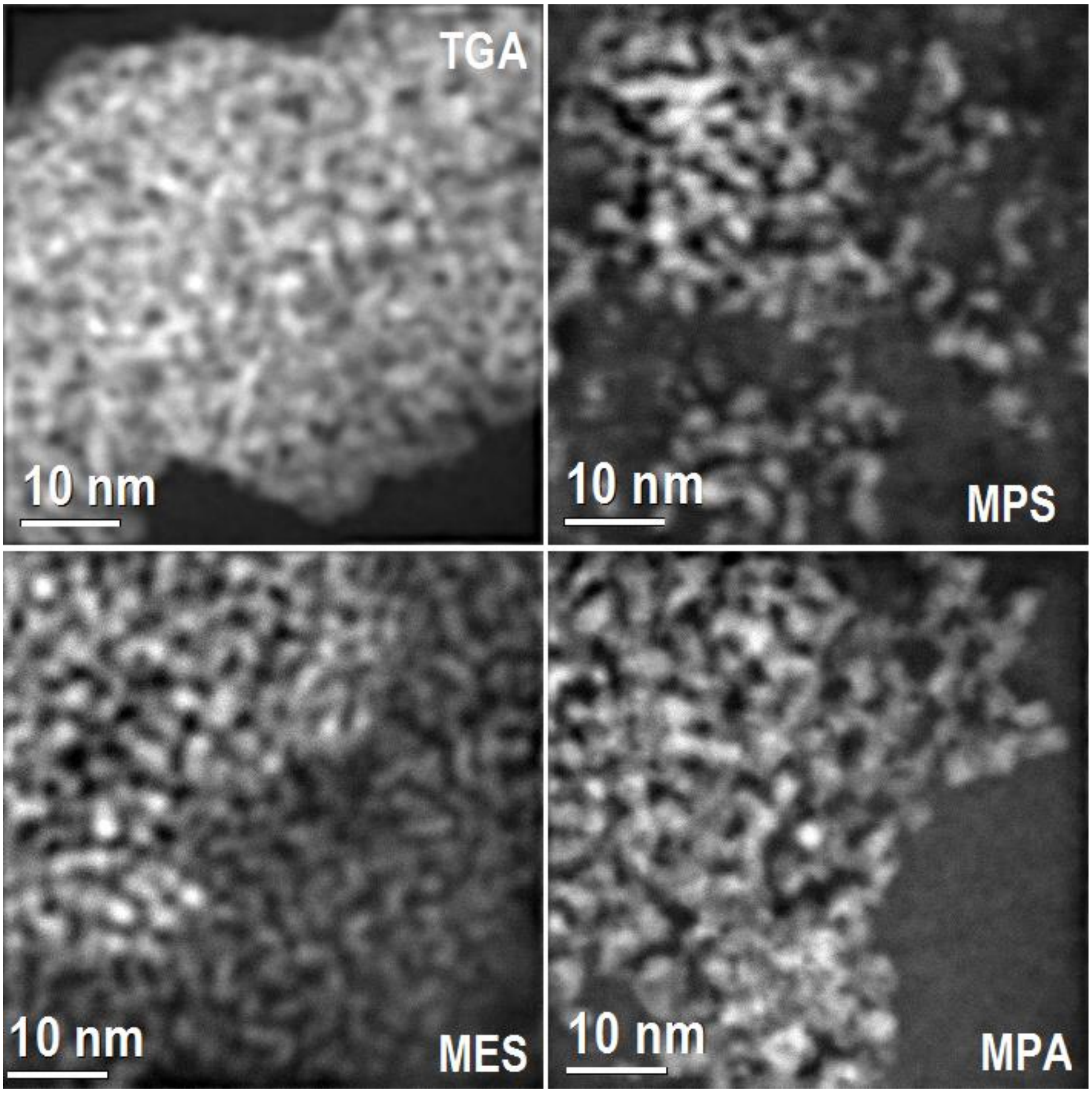

Fig 6S: STEM images of CdTe QDs capped with different SL.

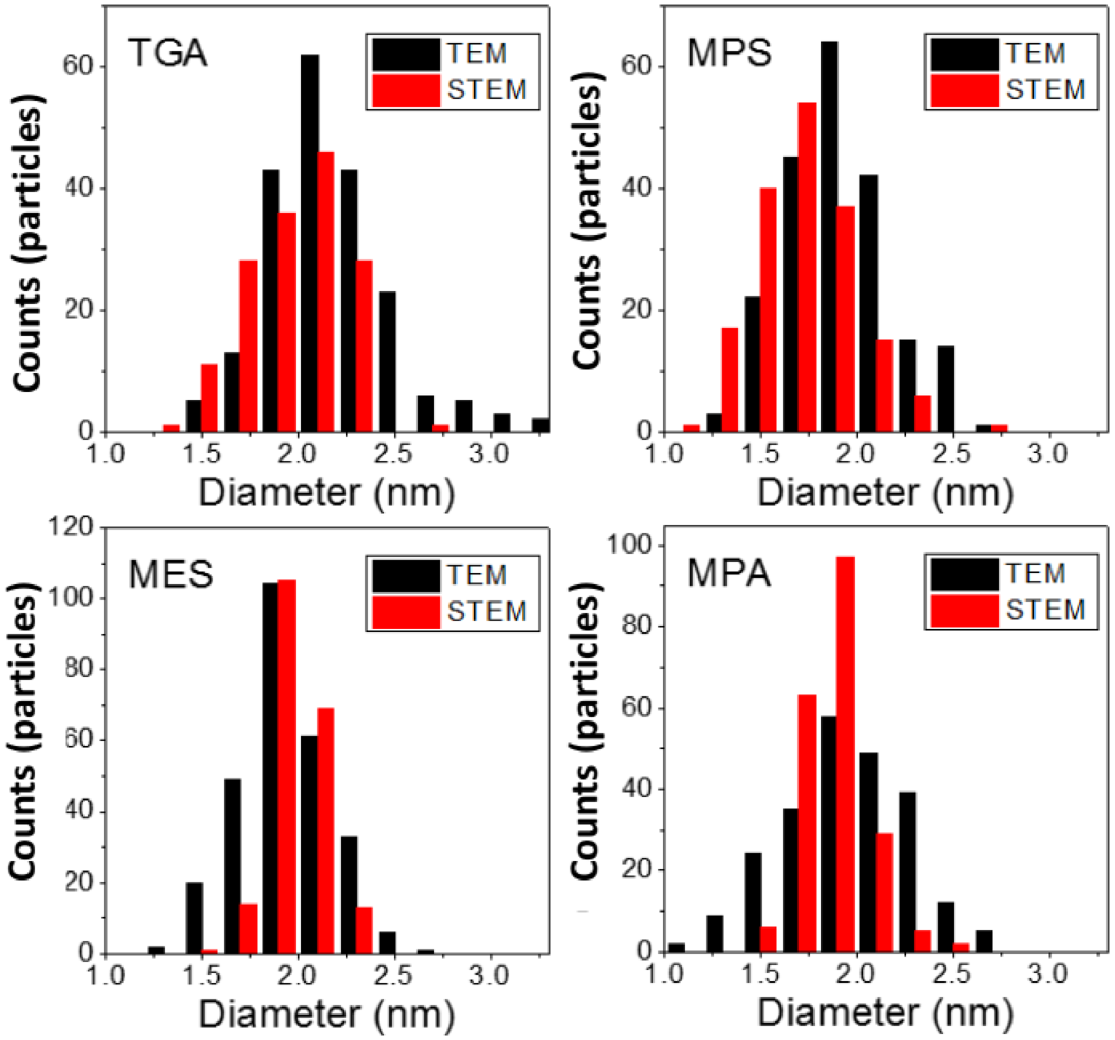

Fig. 7S: Size distribution of CdTe QDs capped with different SL. The size distribution was calculated by TEM and STEM measurements.

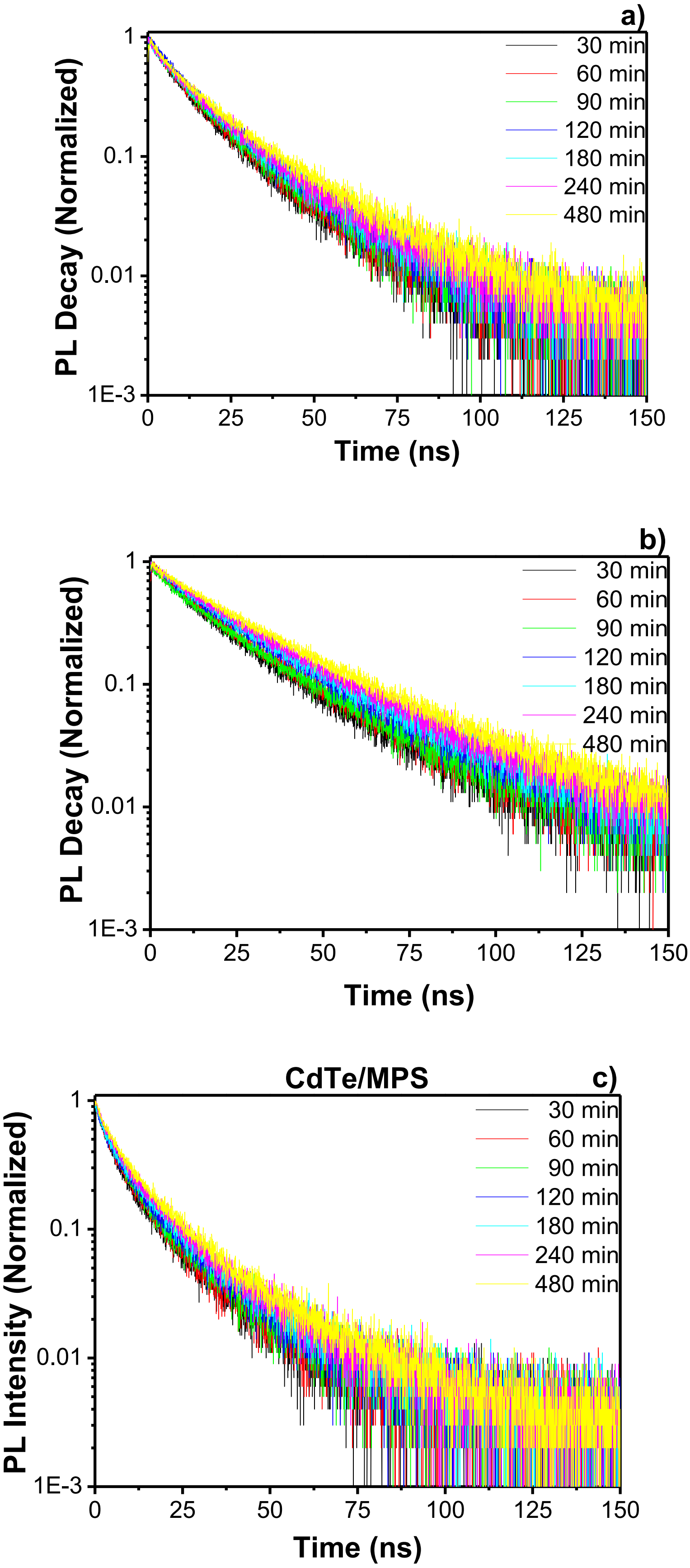

Fig. 8S: PL decay curve of CdTe QDs capped with a) TGA, b) MES and C) MPS ligands.

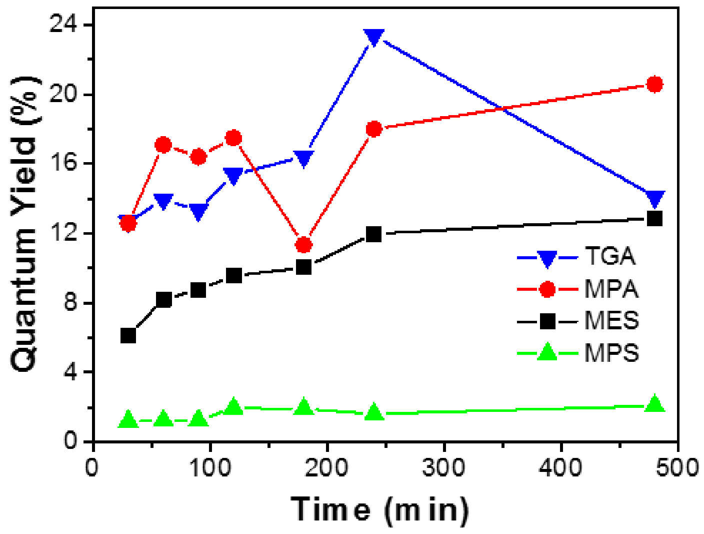

Fig. 9S: PL quantum yield of CdTe QDs capped with different surface ligand in function of synthesis time.

Table 1S. Parameters obtained from PL decay curves of CdTe/MPA QDs

Sample	α_1(%)	τ_1(ns)	α_2(%)	τ_2(ns)	α_1 rel. (%)	α_2 rel. (%)	$\bar{\tau}$ (ns)	χ^2
30 min	67.0	6.6±0.2	33.0	24.4±0.2	35.15	64.85	18.1	1.094
60 min	65.0	6.4±0.2	35.0	23.5±0.2	33.34	66.66	17.8	1.081
90 min	66.0	6.9±0.2	34.0	24.0±0.2	36.04	63.96	17.8	1.114
120 min	65.0	6.8±0.2	35.0	24.1±0.2	34.61	65.39	18.1	1.123
180 min	66.0	7.3±0.2	34.0	25.2±0.2	36.39	63.61	18.7	1.173
240 min	66.0	8.0±0.2	34.0	26.5±0.2	37.23	62.77	19.6	1.063
480 min	62.0	8.8±0.2	38.0	27.2±0.2	34.45	65.55	20.9	1.128

Table 2S: Parameters obtained from PL decay curves of CdTe/TGA QDs

Sample	α_1(%)	τ_1(ns)	α_2(%)	τ_2(ns)	α_1 rel. (%)	α_2 rel. (%)	$\bar{\tau}$ (ns)	χ^2
30 min	63.0	7.9±0.2	37.0	21.2±0.2	38.69	61.31	16.0	1.103
60 min	59.0	8.2 ±0.2	41.0	21.4±0.2	35.62	64.38	16.7	1.126
90 min	60.0	8.5±0.3	40.0	22.1±0.2	36.96	63.04	17.1	1.114
120 min	57.0	8.7±0.3	43.0	22.3±0.2	33.99	66.01	17.7	1.107
180 min	56.0	8.7±0.3	44.0	23.0±0.2	32.36	67.64	18.4	1.179
240 min	55.0	8.9±0.3	45.0	23.4±0.2	31.47	68.53	18.8	1.097
480 min	49.0	8.1±0.3	51.0	24.7±0.2	24.04	75.96	20.7	1.128

Table 3S: Parameters obtained from PL decay curves of CdTe/MES QDs

Sample	α_1(%)	τ_1(ns)	α_2(%)	τ_2(ns)	α_1 rel. (%)	α_2 rel. (%)	$\bar{\tau}$ (ns)	χ^2
30 min	48.0	9.7±0.4	52.0	25.6±0.2	25.52	74.48	21.5	1.118
60 min	40.0	9.7±0.4	60.0	26.1±0.2	19.88	80.12	22.8	1.012
90 min	39.0	10.0±0.5	61.0	26.3±0.2	19.34	80.66	23.1	1.056
120 min	35.0	10.4±0.7	65.0	27.8±0.2	16.74	83.26	24.9	1.039
180 min	39.0	12.5±0.7	61.0	29.5±0.2	21.17	78.83	25.9	1.058
240 min	33.0	12.6±0.5	67.0	30.2±0.2	17.34	82.66	27.1	1.047
480 min	29.0	13.1±0.3	71.0	32.0±0.3	14.47	85.53	29.3	1.030

Table 4S: Parameters obtained from PL decay curves of CdTe/MPS QDs

Sample	α_1(%)	τ_1(ns)	α_2(%)	τ_2(ns)	α_1 rel. (%)	α_2 rel. (%)	$\bar{\tau}$ (ns)	χ^2
30 min	72.0	4.4±0.1	28.0	17.2±0.1	39.70	60.30	12.1	1.215
60 min	71.0	4.4±0.1	29.0	17.4±0.1	38.38	61.62	12.4	1.197
90 min	71.0	4.8±0.1	29.0	18.4±0.2	39.16	60.84	13.1	1.175
120 min	70.0	5.1±0.1	30.0	19.5±0.2	37.20	62.80	14.1	1.186
180 min	67.0	5.1±0.1	33.0	19.8±0.2	33.90	66.10	14.0	1.210
240 min	68.0	5.3±0.1	32.0	20.0±0.1	35.89	64.11	14.7	1.160
480 min	68.0	5.9±0.1	32.0	21.6±0.2	36.74	63.26	15.8	1.212

PL Quantum Yield

The PL quantum yield (Φ_f) can be defined by the number of photons that are emitted by the number of photons that have been absorbed, as shown in equation 1SI.

$$\Phi_f = \frac{N_{em}}{N_{abs}} \quad \text{(Eq. 1SI)}$$

The quantum yield measures the probability of the excited state be disabled by fluorescence phenomenon, loss of energy by the emission of photons, and not by other phenomena not as radiative internal conversion or relaxation vibration.

The method was performed using the comparative method, using a dye with a known quantum yield. The dye used was Rhodamine 6G. The procedure is to record the PL and absorption spectra at the same wavelength that was used to make the excitation in PL measurements, in all cases we used 355 nm. There were measured at least six data points of different absorbance in the range of 0.006-0.05. A graph of the integrated area of PL versus absorbance was plotted and a linear fit of the data was applied.

$$\Phi_x = \Phi_{st}\left(\frac{Slope_x}{Slope_{st}}\right)\left(\frac{\eta_x^2}{\eta_{st}^2}\right) \quad \text{(Eq. 2SI)}$$

where *x* and *st* represent sample and reference standard, respectively. Slope refers to the angular coefficient of the curves obtained, η is the refractive index of the medium, and Φ is the quantum yield.

As the solutions used were very dilute it can be assumed that the refractive index was very close to the pure solvent. As the solvent was the same in all analyses, the term of the second brackets can be simplified to facilitate the calculations.

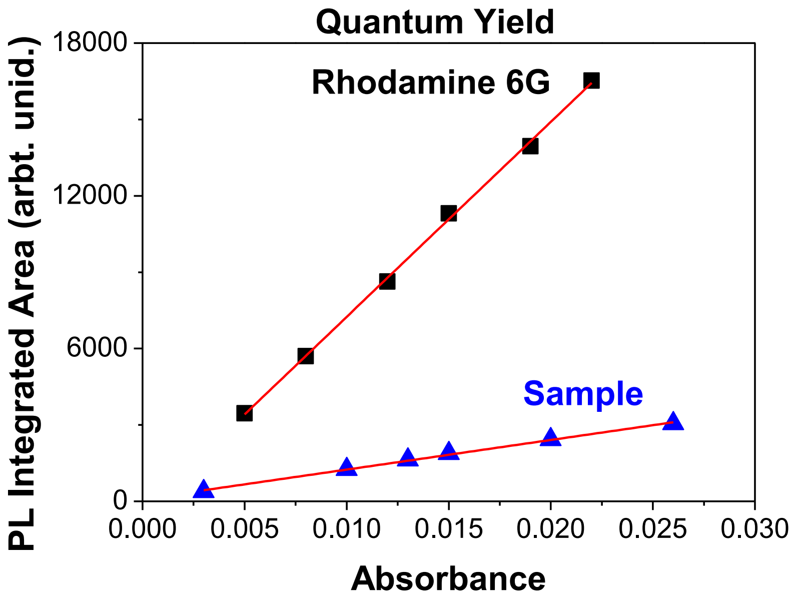

Fig. 10S: Typical curves to determine PL quantum yield.

The Table 5 SI shows the values of slope obtained by fitting of linear equation to fluorescence quantum yield.

Table 5S: Obtained values of slope and PL quantum yield

Sample	Slope	Φ (%)
**CdTe/MPA**		
30 min	1.05±0.04 E5	12.6±0.5
60 min	1.42±0.02 E5	17.1±0.4
90 min	1.37±0.02 E5	16.4±0.3
120 min	1.46±0.03 E5	17.5±0.5
180 min	9.45±0.09 E4	11.3±0.2
240 min	1.50±0.03 E5	18.0±0.4
480 min	1.71±0.06 E5	20.6±0.8
**CdTe/MPS**		
30 min	1.01±0.03 E4	1.21±0.05
60 min	1.05±0.03 E4	1.25±0.05
90 min	1.06±0.03 E4	1.27±0.04
120 min	1.63±0.04 E4	1.96±0.06

180 min	1.60±0.05 E4	1.92±0.06
240 min	1.35±0.04 E4	1.63±0.05
480 min	1.74±0.03 E4	2.10±0.05
**CdTe/TGA**		
30 min	1.06±0.04 E5	12.7±0.5
60 min	1.16±0.03 E5	13.9±0.4
90 min	1.11±0.04 E5	13.3±0.5
120 min	1.28±0.05 E5	15.4±0.6
180 min	1.37±0.05 E5	16.4±0.6
240 min	1.95±0.06 E5	23.4±0.8
480 min	1.17±0.04 E5	14.1 ±0.6
**CdTe/MES**		
30 min	5.10±0.2 E4	6.1±0.2
60 min	6.84±0.3 E4	8.2±0.3
90 min	7.29±0.2 E4	8.8±0.3
120 min	7.98±0.2 E4	9.6±0.3
180 min	8.35±0.3 E4	10.0±0.3
240 min	9.97±0.3 E4	12.0±0.4
480 min	1.07±0.3 E4	12.9±0.4
**Rhodamine 6G***	7.7±0.1 E5	92±3

*The value of PL quantum yield was obtained from literature[1] and the standard deviation was obtained by standard deviation of slope.

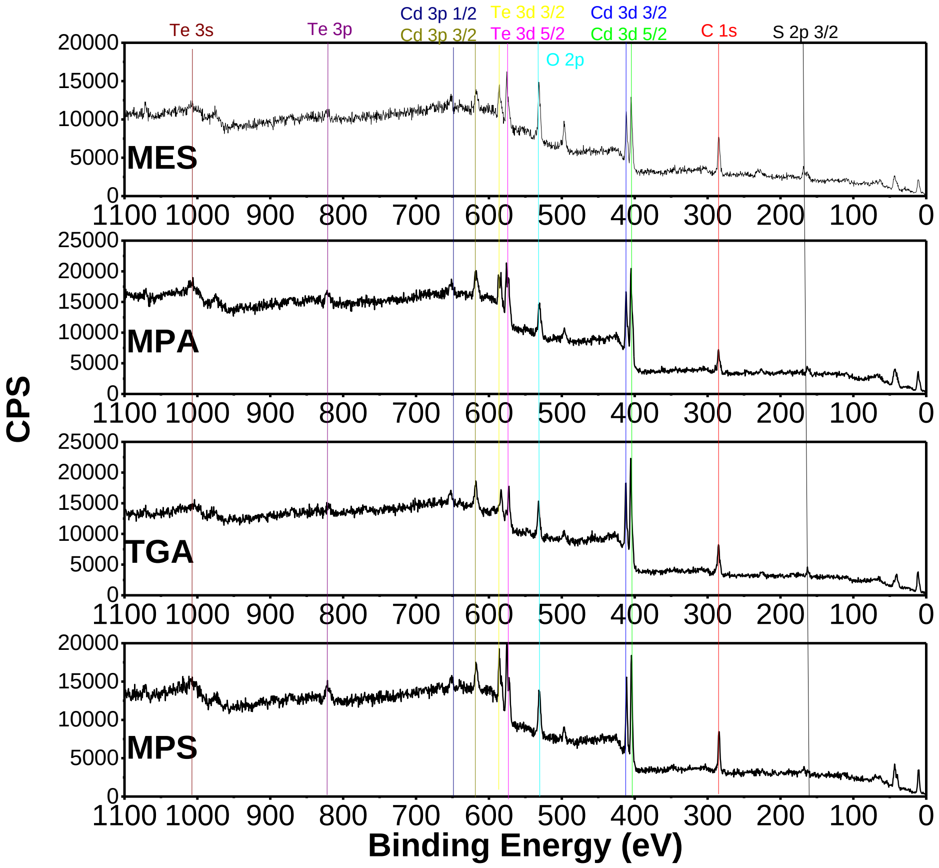

Fig. 11S: Survey XPS spectrum of CdTe/MES, CdTe/MPA, CdTe/TGA and CdTe/MPS

In order to distinguish between the thiolate and sulfide sulfur, which often occur at similar energies we have performed an experiment following [Li et. Al. J. Am. Chem. Soc. 2013, 135, 12270−12278]: the S 2p spectra of CdTe-TGA and CdTe-MPA QD were compared before and after the prolonged X-ray exposure. The study showed that in agreement with the previous studies the peak of the thiolates (at higher energy) decreased in intensity compared to the sulfide peak CdS (at lower energy) likely because of the gradual degradation of the former under the X-ray irradiation.

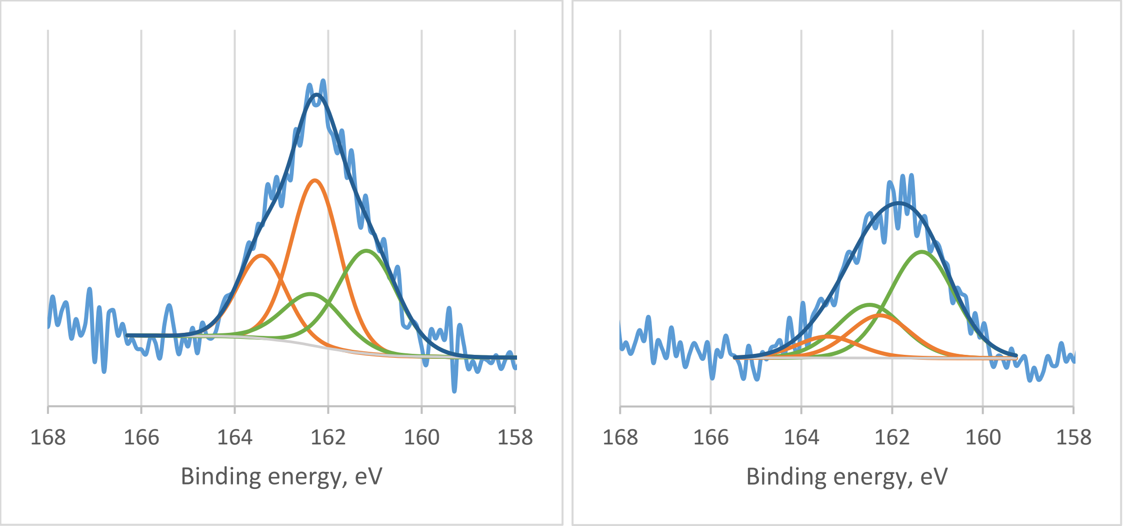

Figure 12S. S 2p XPS peak of CdTe-TGA QDs before (left) and after (right) exposure to the X-ray beam for 90 minutes. A doublet at higher binding energy (orange curves) has decreased in intenssity compared to the sulfide peak (green curves).

Table 6S. Total ratios between the key elements of CdTe QDs determined by EDX and XPS.

	EDX			XPS		
	Te/Cd	Cd/S	Te/S	Te/Cd	Cd/S	Te/S
TGA	0.57	1.55	0.88	0.34	1.58	0.55
MPA	0.64	1.72	1.11	0.61	1.69	1.03
MES	0.77	0.97	0.75	0.60	0.53	0.32
MPS	0.80	0.93	0.74	0.72	1.28	0.92

As an asymmetry in the emission spectra of QD, particularly in those capped with MES and MPS, was observed, it was decided to fit the peaks with several Gaussian functions, in order to better understand such asymmetry phenomenon. The results are shown in the Figures 9 SI to 12 SI.

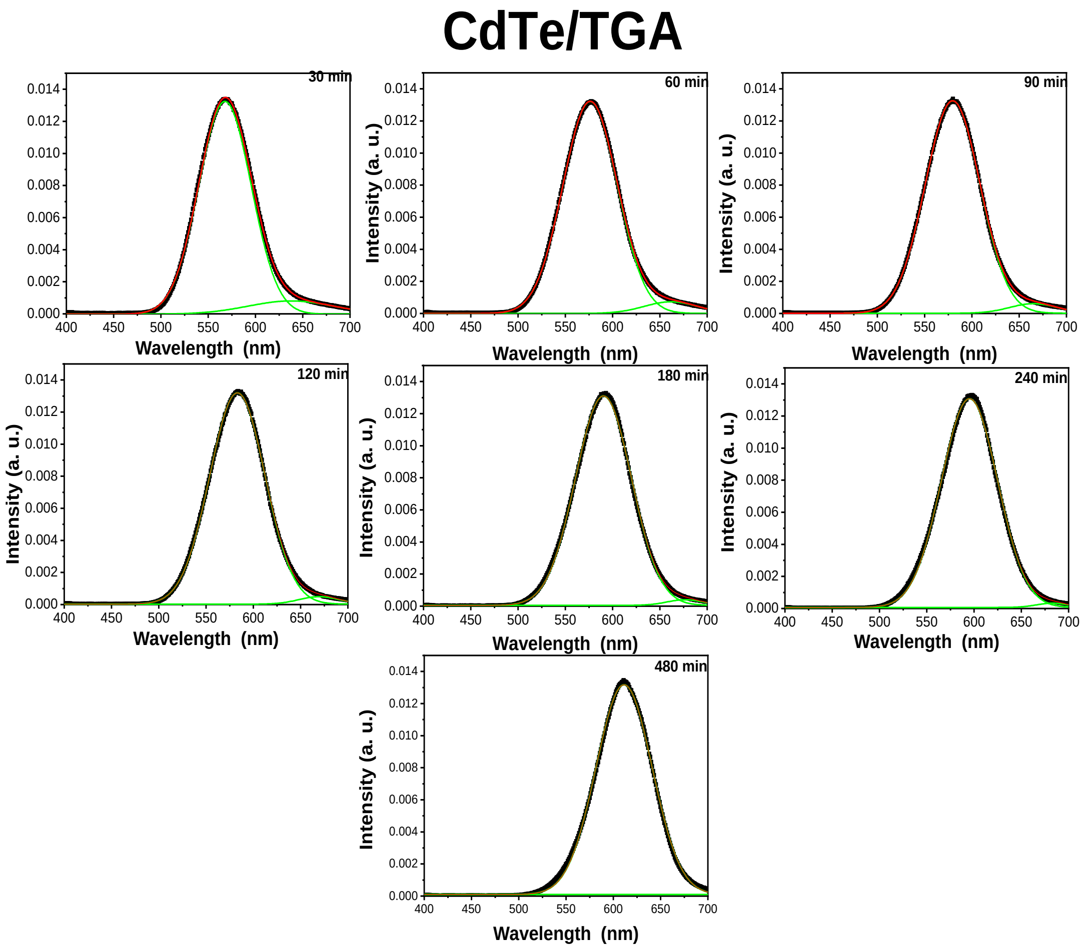

Fig. 13S: Deconvoluted emission spectra of CdTe/TGA.

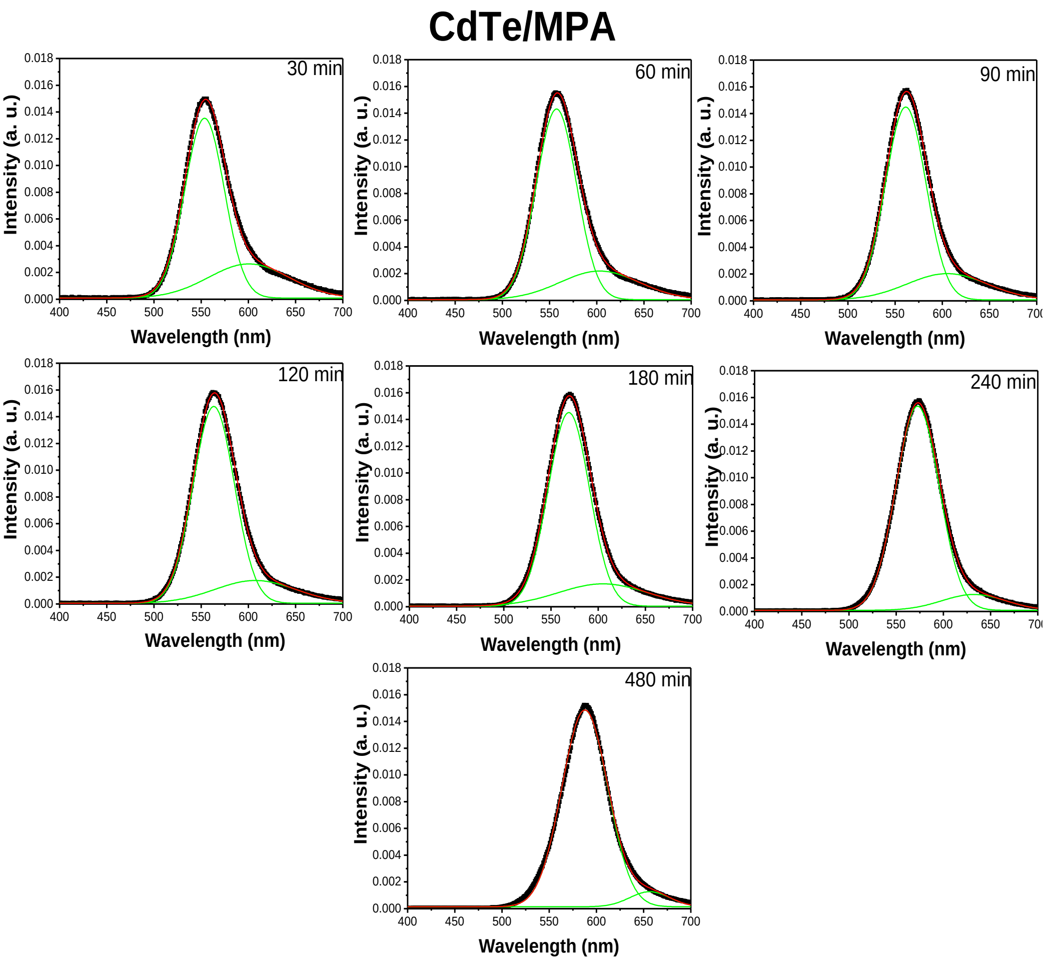

Fig. 14S: Deconvoluted emission spectra of CdTe/MPA.

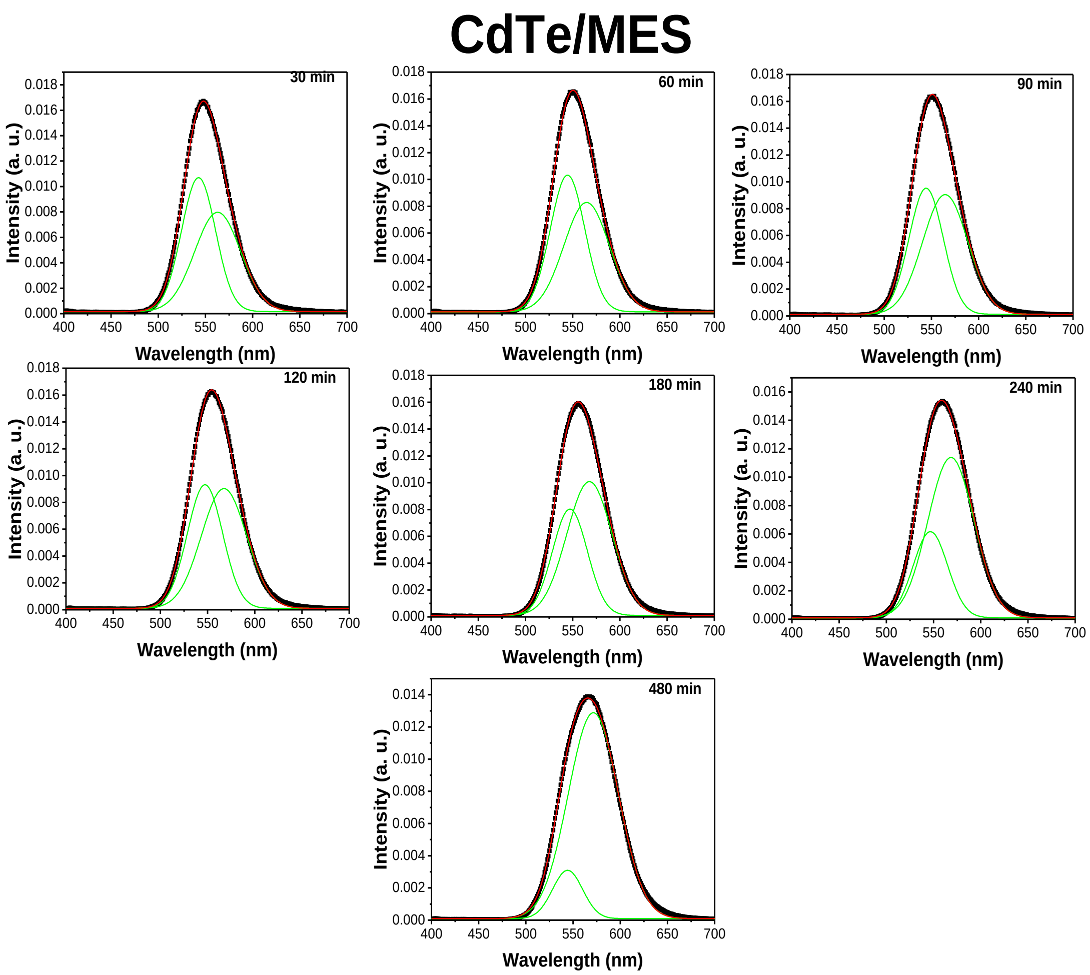

Fig. 15S: Deconvoluted emission spectra of CdTe/MES.

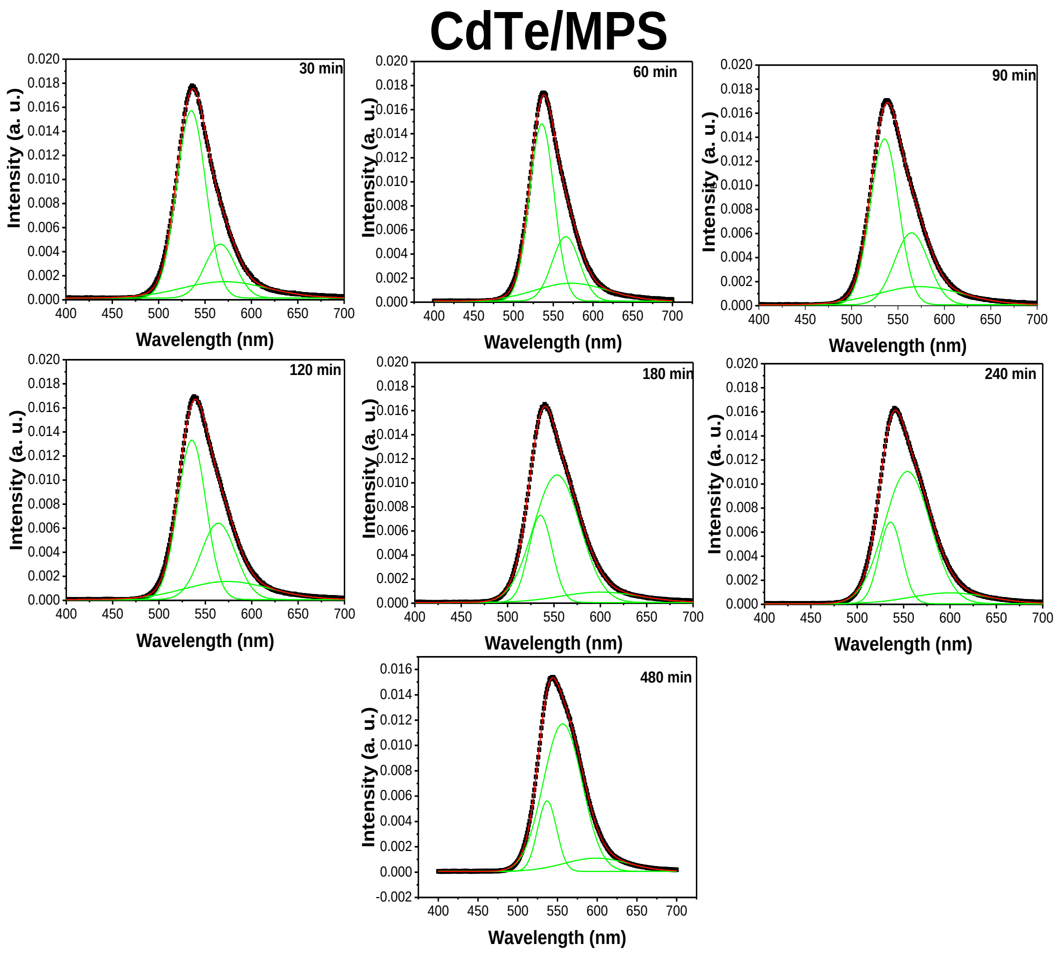

Fig. 16S: Deconvoluted emission spectra of CdTe/MPS.

The deconvolution results for CdTe/TGA and CdTe/MPA are quite similar with two bands, one due to exciton recombination and another one due to shallow trap states, which possibly originate from TeO_2, as suggested by XPS results. The bands located at low energy for CdTe/TGA are much less intense and are located at lower energies than those of CdTe/MPA, and both diminish with synthesis time in agreement with the literature data and all results presented here. As a consequence, we hypothesize that higher concentration of the traps increases the energy level of trap states. CdTe/MES PL also shows two bands, however the band located at lower energy is shifted compared to the first one only by 80 meV, while for TGA and MPA ligands the observed shift is of 270 and 200 meV, respectively. On the other hand, the PL signal of CdTe/MPS QDs shows three bands shifted by 75 and 250 meV. This energy difference suggests that the type of the traps in TGA and MPA-capped QDs is different from MES ones, while in the case of MPS there are likely two types of trap states. Taking into account the XPS and PL lifetime results, we can conclude that the CdTe/MPA and CdTe/TGA QDs formed a

CdTe/CdS core/shell alignment of type I. In this situation, the energy level of the shell doesn't contribute to the emission spectra, once it is higher than the bottom of the CB of the core. Because of that, the band located ~200 meV in this configuration can be attributed to emissive trap states. CdTe/MES QDs formed a CdTe/Cd(TeO_2)S core/shell alignment quasi-type II. Here the CB of the core and shell is quite close, because of that the two bands in the emission spectra can be attributed to the recombination in the core and in the shell. The increase of the amplitude of the longer component in the PL lifetime for CdTe/MES QDs is in accord to our deconvoluted emission spectra, see Figure 17 S. Finally, CdTe/MPS formed CdTe/$CdTeO_2$ inverted type I alignment, and the three bands can be attributed to the recombination in the core, shell, and trap states. Moreover, we have notice that the maximum of the absorption band and emission band of the CdTe/MPS QDs is completely different from the other systems studied here, Figure 4 S. CdTe/TGA QD showed redshift about 40 nm in both absorption and emission spectra, CdTe/MPA ~30 nm, and CdTe/MES ~10 nm. However, CdTe/MPS showed ~30 nm in the absorption spectra, and just ~5 nm in the emission spectra. This result is odd, it indicates that the core grown up, and the emission is not governed by the core, but by the shell, which confirm our previous conclusion about the inverted-type I alignment.

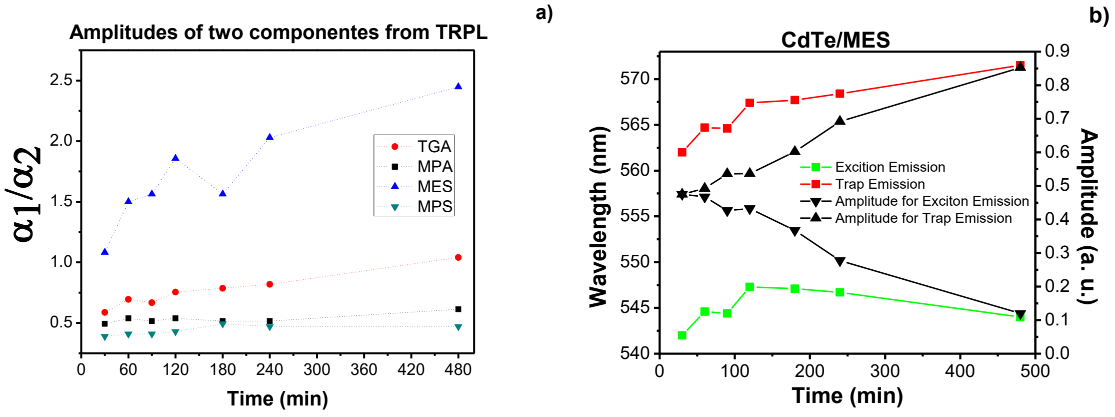

Fig. 17S: a) Amplitude ratio extracted from time-resolved PL and b) Data extracted from deconvoluted emission spectra of CdTe/MES.

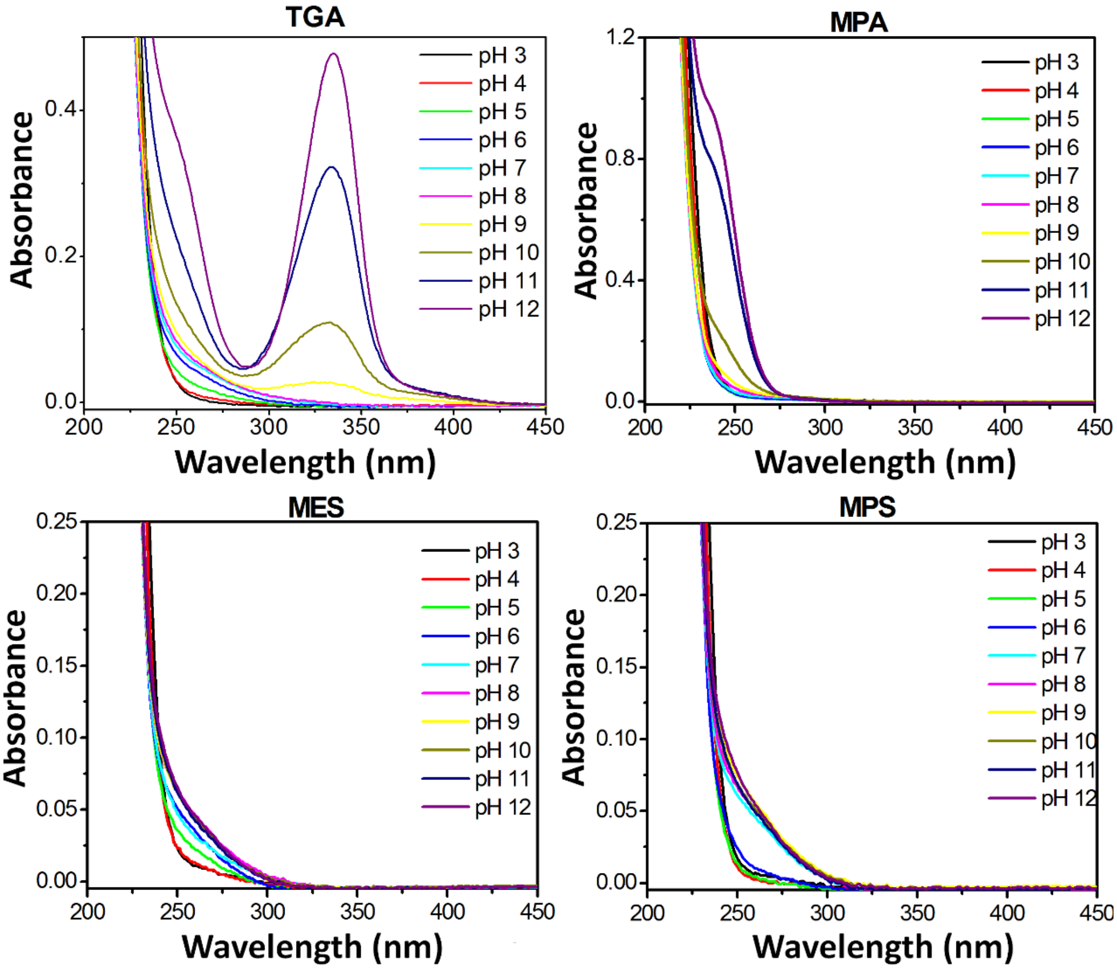

Figure 18S. UV-Vis Absorption spectra of ligand solution a) TGA, b) MPA, c) MES and d) MPS at diverse pH.

Calculation of the number of atoms on the surface of the QDs

This calculation was used as an approximation based just in the diameter of the QD, properties of the CdTe and geometry of the physic system.[2] We take the unit cell of the CdTe (zinc blend) which has 0.6482 nm of lattice parameter, volume = $(0.6482\ nm)^3$ = $(0.2723 \times 10^{-27}\ m^3)$, 5870 kg m^{-3} of density, 240 g/mol molar mass, 1.594×10^{-21} g is the mass of one unit cell (m'). Number of atoms inside of zinc blend unit cell is equal to 8. The volume of the sphere is $(4/3)*\pi r^3$. Then, with these parameters we will be able to calculate the total number of atoms, in the core, and around the surface of the QD.

Number of unit cells which consist the QD = mass/ Molar Weight => n = m/MW

Density = mass/volume => d = m/v => m = d*v

$n = d*V_{QD}/m'$ (3SI)

$V_{QD} = (4/3)*\pi r^3_{qd} = 4{,}1888\ nm^3$ (taking the diameter of the CdTe equal to 2 nm, according to Fig 2a main text)

Solving Equation 3SI and using the result for the volume of each QD is equal to the number of unit cells which consist the QD.

$n_{QD} = d*V_{QD}/m'$ => 15.43 unit cells

Considering there are 8 atoms inside of each unit cells (n_i), then $n_t = 8*\ n_{QD} = 123.4$ atoms.

However, this result is for all atoms in the QD. In order to know the number of unit cells just inside (in the core) of the QD, we have to subtracted by the atoms from the surface, and it will give the core volume (V_c), which is the radii of the QDs less the nearest atom neighbor (α). α is 0.281 nm for CdTe in zinc blend structure.

$V_c = 4/3*\pi*(r- \alpha)^3 = 1.557\ nm^3$

Finally, it's possible to know the number of unit cells of the core (n_c) and total number of atoms in the core (n_{ct}).

$n_c = d*V_s/m' = 5{,}733$ unit cells => $n_{ct} = 8*d*V_s/m' = 45.87$ atoms

So, the number of atoms around the surface (n_s) is the total number of atoms of the QDs less the number of atoms in the core.

$n_s = n_t – n_{ct} = 123.4 – 45.87 = 77.53$ atoms.

$\% = (n_s/n_t)*100\% = 62.8\% \approx 63\%$.

References

1. Brouwer, A. M., Standards for photoluminescence quantum yield measurements in solution (IUPAC Technical Report). *Pure and Applied Chemistry* **2011,** *83* (12).

2. P. Reis, M. et al, Core/Shell Semiconductor Nanocrytals, Small **2009**, 2, 154.
